# Supplementary material for: Do socioeconomic inequities arise during school-based physical activity interventions? An exploratory case study of the GoActive trial
Source: BMJ Open. 2023 Mar 13;13(3):e065953. doi: 10.1136/bmjopen-2022-065953 (PMC10016273; doi:10.1136/bmjopen-2022-065953)
Supplement: Supplementary data [file bmjopen-2022-065953supp001.pdf]

Supplementary Table 1: Mean physical activity and anthropometric outcomes by socioeconomic position and randomisation group at each measurement point

	**Baseline**		**14-16 weeks post intervention**		**10-months post intervention**	
	Intervention	Control	Intervention	Control	Intervention	Control
**Low-SEP** *Mean (SD)*						
MVPA (minutes)	34.1 (15.4)	36.0 (20.9)	35.6 (21.0)	36.2 (21.7)	29.7 (24.0)	27.4 (17.5)
BMI z-Score	0.26 (2.06)	0.41 (1.71)			0.32 (1.93)	0.64 (1.33)
Body fat (%)	22.0 (9.6)	22.3 (11.1)			18.5 (12.8)	20.6 (12.9)
Waist circumference (cm)	71.2 (10.2)	72.5 (12.5)			72.4 (14.5)	74.8 (12.3)
**Middle/high-SEP** *Mean (SD)*						
MVPA (minutes)	35.9 (18.7)	35.5 (18.7)	33.3 (22.3)	35.4 (21.4)	25.0 (21.0)	27.7 (20.9)
BMI z-Score	0.10 (1.90)	0.17 (1.63)			0.10 (1.88)	0.15 (1.76)
Body fat (%)	20.7 (9.9)	20.5 (9.9)			18.2 (11.7)	18.6 (11.5)
Waist circumference (cm)	70.2 (9.5)	69.7 (9.1)			70.4 (14.3)	70.7 (13.1)

SEP=Socioeconomic position, SD=Standard deviation, MVPA=Moderate-to-vigorous physical activity, BMI=Body mass index
