# Supplementary material for: Do socioeconomic inequities arise during school-based physical activity interventions? An exploratory case study of the GoActive trial
Source: BMJ Open. 2023 Mar 13;13(3):e065953. doi: 10.1136/bmjopen-2022-065953 (PMC10016273; doi:10.1136/bmjopen-2022-065953)
Supplement: Author's manuscript [file bmjopen-2022-065953.draft_revisions.pdf]

BMJ Open

BMJ Open is committed to open peer review. As part of this commitment we make the peer review history of every article we publish publicly available.

When an article is published we post the peer reviewers' comments and the authors' responses online. We also post the versions of the paper that were used during peer review. These are the versions that the peer review comments apply to.

The versions of the paper that follow are the versions that were submitted during the peer review process. They are not the versions of record or the final published versions. They should not be cited or distributed as the published version of this manuscript.

BMJ Open is an open access journal and the full, final, typeset and author-corrected version of record of the manuscript is available on our site with no access controls, subscription charges or pay-per-view fees (http://bmjopen.bmj.com).

If you have any questions on BMJ Open's open peer review process please email info.bmjopen@bmj.com

BMJ Open

Do socioeconomic inequalities arise during school-based physical activity interventions? An exploratory case study of the GoActive trial

Journal:	*BMJ Open*
Manuscript ID	bmjopen-2022-065953
Article Type:	Original research
Date Submitted by the Author:	31-Jul-2022
Complete List of Authors:	Alliott, Olivia; University of Cambridge, MRC Epidemiology Unit<br>Fairbrother , Hannah; University of Sheffield, Health Sciences School<br>Corder, Kirsten ; University of Cambridge, MRC Epidemiology Unit<br>Wilkinson, Paul; University of Cambridge, Cambridge Neuroscience<br>van Sluijs, Esther; University of Cambridge, MRC Epidemiology Unit
Keywords:	EPIDEMIOLOGY, PUBLIC HEALTH, Community child health < PAEDIATRICS

SCHOLARONE™
Manuscripts

I, the Submitting Author has the right to grant and does grant on behalf of all authors of the Work (as defined in the below author licence), an exclusive licence and/or a non-exclusive licence for contributions from authors who are: i) UK Crown employees; ii) where BMJ has agreed a CC-BY licence shall apply, and/or iii) in accordance with the terms applicable for US Federal Government officers or employees acting as part of their official duties; on a worldwide, perpetual, irrevocable, royalty-free basis to BMJ Publishing Group Ltd ("BMJ") its licensees and where the relevant Journal is co-owned by BMJ to the co-owners of the Journal, to publish the Work in this journal and any other BMJ products and to exploit all rights, as set out in our licence.

The Submitting Author accepts and understands that any supply made under these terms is made by BMJ to the Submitting Author unless you are acting as an employee on behalf of your employer or a postgraduate student of an affiliated institution which is paying any applicable article publishing charge ("APC") for Open Access articles. Where the Submitting Author wishes to make the Work available on an Open Access basis (and intends to pay the relevant APC), the terms of reuse of such Open Access shall be governed by a Creative Commons licence – details of these licences and which Creative Commons licence will apply to this Work are set out in our licence referred to above.

Other than as permitted in any relevant BMJ Author's Self Archiving Policies, I confirm this Work has not been accepted for publication elsewhere, is not being considered for publication elsewhere and does not duplicate material already published. I confirm all authors consent to publication of this Work and authorise the granting of this licence.

For peer review only

Do socioeconomic inequalities arise during school-based physical activity interventions? An exploratory case study of the GoActive trial

Olivia Alliott[1], Hannah Fairbrother[2], Kirsten Corder[1], Paul Wilkinson[3] & Esther van Sluijs[1]

1.MRC Epidemiology Unit, University of Cambridge School of Clinical Medicine, Cambridge, UK

2. Health Sciences School, University of Sheffield, UK

3. Department of Psychiatry, University of Cambridge, Cambridge, UK

Corresponding Author

Dr Esther van Sluijs, University of Cambridge

Email: esther.vansluijs@mrc-epid.cam.ac.uk

Phone: +44 (0) 1223 769189

For peer review only

Abstract

Objective To investigate potential socioeconomic inequalities in the implementation and evaluation of the GoActive school-based physical activity intervention and demonstrate a novel way of evaluating intervention-related inequalities.

Study design Secondary analysis of trial data

Setting Secondary schools across Cambridgeshire and Essex, UK

Participants 13-14-year-old adolescents (n=2838, 16 schools)

Methods Socioeconomic inequalities across 6 stages in the intervention and research process were evaluated: (1) provision of and access to intervention resources (2) intervention uptake (3) intervention effectiveness (accelerometer-assessed moderate-to-vigorous physical activity (MVPA)) (4) long-term compliance (5) response in evaluation (6) impact on health. Data from self-report and objective measures were analysed by individual- and school-level socioeconomic position (SEP) using a combination of classical hypothesis tests and multilevel regression modelling.

Results Stage: (1) There was no difference in the provision of physical activity resources by school-level SEP. (2) There was no difference in initial intervention uptake, but students of low-SEP engaged significantly less with the intervention (proportion accessing intervention website: Low=37.2%; Middle=45.4%; high=47.0%; p=0.001). (3) Interaction analyses suggest a positive intervention effect on MVPA in adolescents of low-SEP (3.13 minutes/day; 95%CI: -1.27,7.54, but not middle/high (-1.49; -6.54,3.57). (4) At 10-months post intervention, this difference increased and reached statistical significance (low SEP: 4.90; 0.09,9.70; middle/high SEP: -2.76; -6.78,1.26). (5) There was greater non-compliance to evaluation measures among adolescents of low-SEP (% non-compliance (low vs. high SEP): baseline: 11.6 vs. 7.5; post-intervention: 38.4 vs. 30.8; follow-up: 45.5 vs. 29.8). (6) The intervention effect

For peer review only

on BMI z-score was more favourable in adolescents of low-SEP (Low SEP: -0.10; -0.19,0.00, middle/high: 0.03; -0.047,0.12).

Conclusions These novel exploratory analyses suggest that the GoActive intervention had a more favourable positive effect on MVPA and BMI in adolescents of low-SEP, despite lower intervention engagement. However, differential response to evaluation measures may have biased these conclusions. We demonstrate a novel way of evaluating inequalities within young people's physical activity intervention evaluations.

Strengths and limitations of this study

- We present a novel approach to evaluating inequalities throughout the intervention process of young people's physical activity interventions. This approach provides additional insight for the development of effective and equitable interventions.
- Whilst exploratory rather than confirmatory, the small sample size of the low-SEP group raises problems with regard to statistical power. Further limitations include the low number of schools.
- The conclusions may therefore be affected by attrition bias, as attrition was largest among participants of a low-SEP.

For peer review only

1. Introduction

The health benefits of physical activity are well-established[1] and physical inactivity has been identified as a major public health concern.[2] Physical inactivity in adolescence is linked to poor health and has long-lasting implications for health and life chances throughout adulthood.[3] However, globally over 80% of students aged 11-17 years are insufficiently active to accrue the benefits.[4]

Similar to other health behaviours, disparities in physical activity during adolescence may contribute to inequalities in current and future health.[5] Recent review-level evidence highlights the importance of promoting and enabling physical activity among adolescents living in the context of socioeconomic deprivation, who report experiencing more barriers to physical activity when compared to other socioeconomic groups.[6] Despite regularly collecting relevant information at baseline, most controlled trials of physical activity interventions in young people do not analyse differences in intervention effect across socioeconomic groups.[7] This has led to a scarcity of evidence regarding whether certain socioeconomic groups may benefit from, or be disadvantaged by, current intervention efforts.[7]

Public health literature suggests the extent to which inequalities are perpetuated or reduced can depend on the nature of the intervention.[8] 'High-risk strategies' target individuals with a higher risk of developing the disease, whereas population strategies attempt to lower the risk of the entire population by shifting the distribution of underlying risk factors, such as physical inactivity.[9] As a consequence of compulsory education in many countries, the potential for schools to deliver wide-reaching and equitable physical activity interventions has been well documented.[10,11] Taking a population approach, school-based interventions have been studied and deemed successful if average physical activity levels increase.[12] However, population strategies have the potential to inadvertently exacerbate health inequalities within a population.[13]

For peer review only

Researchers have begun to consider the potential for interventions to have a differential effect across individuals, commonly named 'intervention generated inequalities.'[8] However, these studies have tended to focus on differential effects by gender.[7] Limited evidence from individual evaluations of school-based interventions and physical activity interventions document socioeconomic inequalities in the provision of, and access to, interventions and resources,[14,15] variation in intervention uptake,[16] differential intervention efficacy,[15,17] differential long-term compliance[18] and differential response in evaluations.[7,19,20]

These previous studies offer examples of various points in the research and intervention process where inequalities might emerge. Going forward we propose a broader approach is needed, looking at intervention generated inequalities throughout the whole research and intervention process of a single intervention. Based on the work of White et al., (2009),[8] Love (2019) identifies key stages throughout the physical activity intervention where inequalities can be introduced. A visual representation of these stages can be seen in Figure 1.[20] Understanding how inequalities might emerge at each of these stages is essential for the development of equitable school-based physical activity interventions, as whilst inequalities at each of these stages could be small, together they may lead to significant inequalities in final outcomes.[8]

The aim of this paper is to take a case-study approach to investigate if and how socioeconomic inequalities arise during the implementation and evaluation of the GoActive school-based physical activity intervention. In doing so, we demonstrate a novel way of studying inequalities across the intervention implementation and evaluation process that could be applied more broadly.

Insert figure 1

For peer review only

2. Methods

This paper describes secondary analyses of the GoActive trial data. Ethical approval for the GoActive trial was obtained from the University of Cambridge Psychology Research Ethics Committee (PRE.2015.126). The trial was prospectively registered (ISRCTN31583496).

2.1 Participants and randomisation

Sixteen state-run secondary schools in Cambridgeshire and Essex agreed to participate. All Year 9 students (age 13-14-years) and their parents/carers received written study information. Students provided written assent and parents provided passive informed consent (opt-out consent).[21] School-level randomisation, stratified by the percentage of students eligible for Pupil Premium funding at each school (below or above county-specific median) and county (Cambridgeshire or Essex), occurred after baseline measurement.[21] Pupil premium funding aims to reduce the effects of deprivation on educational attainment and is used here as a proxy measure for school-level deprivation.[22]

2.2 GoActive intervention

GoActive was a theory-based intervention developed following an evidence-based iterative approach.[21] The primary aim of GoActive was to increase students' moderate-to-vigorous intensity physical activity (MVPA) across the week.[21] GoActive was delivered over 12 weeks to all students in the intervention schools irrespective of whether they participated in study measurements. The control schools followed normal practice.

GoActive was implemented using a tiered-leadership system led by mentors (older students within the school) and supported by peer-elected Year 9 leaders.[21] During the intervention, Year 9 tutor groups chose two activities per week from a selection of twenty. These activities required little or no equipment and were designed to appeal to a variety of students (including Ultimate Frisbee, Zumba and Hula Hoop).

For peer review only

Schools had access to the GoActive intervention website where they could access activity instructions cards which included an overview of each activity, suggested adaptations, safety tips, ‘factoids’ and a short video.[21] Mentors remained with the class throughout the intervention, whereas peer-leaders changed each week. During the first six weeks, additional leadership was provided by a local authority-funded intervention facilitator (health trainers employed by local councils) who continued to provide remote support thereafter.[21]

Teachers were encouraged to dedicate one tutor time per week to do one of the chosen activities as a class. Students could gain points for trying these new activities at any time in or out of school, irrespective of intensity or duration.[21] Students were encouraged to regularly log these points on the GoActive website to unlock rewards such as a sports bag, t-shirt, or hoodie. Individual points remained private; however, these points were entered into between-class competitions.[21]

The results of the main GoActive trail analysis reported no overall intervention effect on average daily MVPA.[23] Subgroup analyses reported a suggestion of a positive intervention effect among students of a low/middle-SEP. Across all MVPA outcomes, those of high-SEP appeared to benefit least when compared to low/middle-SEP students. Full details of the trial methods have been published elsewhere,[21] a brief summary is provided below.

2.3 Methodological approach to the current study

As outlined above, we take a case study approach to demonstrate how socioeconomic inequalities can be assessed throughout the research and intervention process, using the GoActive intervention as an example. To achieve this, we operationalised the model proposed by Love (2019) to include research questions based on the available GoActive data (Figure 2).[20] For the remainder of this paper, we refer to the stages outlined in figure two when describing our research and findings.

This paper focuses on socioeconomic inequalities, therefore all of the research questions presented in figure 2 consider SEP. We use individual- and area-level SEP,

For peer review only

as using these different levels are important when evaluating the full contribution of socioeconomic conditions.[24] The relevance of different indicators of SEP is dependent upon on the research focus, health outcome and stage in the life course.[24] Taking this approach, we used pupil premium funding (see description in section 2.1) as a school-level indicator of SEP during stages 1 and 2, where the object of analysis is the school, not the individual. For the remaining stages, we use an individual-level indicator of SEP derived from the Family Affluence Scale (FAS).[25] In response to recent review evidence[6] and because of our focus on socioeconomic inequities we compare students of low-SEP to students of middle/high-SEP during stages 3, 4 and 5. This is a different approach to that of the main trail which grouped students of low- and middle-SEP together. All measures are described in further detail below.

Insert figure 2

2.4 Measures

Study measurements were taken at four time points during the GoActive trial: Baseline (BL), mid-intervention (T2; 6 weeks after intervention start, post-intervention (T3; 14–16 weeks after intervention start), and 10-month follow-up (T4; 10 months after intervention end.[23]

A summary of demographic measures and the measures specific to each stage are described below. For conciseness, the following shorter titles are applied to each stage: stage 1 – provision and access, stage 2 - intervention uptake, stage 3 – intervention effect, stage 4 – long-term compliance, stage 5 – evaluation participation, stage 6 – health outcomes

2.4.1 Demographic measures

For peer review only

Participant descriptive characteristics were self-reported at baseline.[21] Participants reported gender from 3 response options (male, female, prefer not to say).[23] Individual-level SEP was reported using the FAS, which is composed of six items relating to: (1) family car ownership, (2) holidays, (3) computers, (4) availability of bathrooms, (5) dishwasher ownership, and (6) having their own bedroom. These were used as a proxy measure of individual-level socioeconomic position by summing the answers (possible range 0–13), and dividing into predefined affluence groups (low = 0–6, middle = 7–9, high = 10–13).[23] Ethnicity was self-reported by participants who were given 20 response options and an additional free text option.[23] The reported options were recoded into five categories in accordance with published recommendations: (1) 'white', (2) 'mixed ethnicity' (i.e. identifying with multiple ethnicities), (3) 'Asian' (including South Asian and Chinese), (4) 'African and/or Caribbean' and (5) 'other'.[23,26]

2.4.3 Stage 1-Provision and access

Data on the school physical activity policy and social and physical environment were self-reported at baseline by contact teachers at all schools.[21] These data were collected using a previously used questionnaire.[27]

The questionnaire assessed the provision of and access to intervention resources at baseline. A list of 16 physical activity facilities available at each school were given a quality rating (0=facility not present, 1= low quality facility, 2=middle quality facility, and 3=high quality facility). Ratings were summed and divided by the number of available facilities to give an average quality rating. An average rating was also used to indicate the suitability of the school grounds for sport, informal games and general play across three measures (1=not at all suitable, 2=somewhat suitable and 3=very suitable). The extracurricular opportunities on offer at each school were derived from a list of 24 (including space to add 'other' activities; one activity = one point e.g. Rounders = 1). Weekly hours of PE were measured using an open-ended question, where teachers rounded to the nearest half-hour. The suitability of the area around the school for physical activity was assessed on a scale of 1-5 (1=strongly disagree to

For peer review only

5=strongly agree) across three measures, shielding from hedges/tress/fences, maintenance of the grounds and the presence of vandalism. Finally, the school's attitude towards physical activity was assessed using the same 1-5 agreement scale across five measures which included encouraging physical activity at school and outside school, educating about the risks of physical activity and how to practice safe physical activity and encouraging active travel.

Pupil premium was used as a school-level indicator of SEP, which was reported by teachers in the school environment questionnaire.[23]

2.4.4 Stage 2- Intervention uptake

Under stage 2, research questions explore engagement with the GoActive intervention. Recruitment data were used to assess the initial uptake of the intervention by school-level SEP. Evaluation uptake was measured as whether participants provided baseline questionnaire data, which was a requirement for participating in GoActive.[23] Trained measurement staff checked the questionnaires upon completion and helped students complete missing sections.[21] Intervention uptake was assessed using data on students' engagement with the GoActive website. This included whether students accessed the GoActive website at any time during the intervention period and was recorded as a categorical variable (accessed vs not). Of the students who did access the website, the number of times they visited and the number of points they logged throughout the intervention were recorded.

2.4.5 Stage 3- Intervention effect

During stage 3, differential intervention efficacy was explored for the GoActive primary outcome, daily accelerometer assessed MVPA at 14-16 weeks post-intervention.[21] Participants were asked to wear a wrist-worn activity monitor (Axivity) assessing acceleration (continuous waveform data) continuously (24 hours a day) for 7 days.[21] The axivity monitor has been validated to assess energy expenditure and to have increased wear time adherence and acceptability than hip-worn monitors in

For peer review only

adolescents.[21,28-30] Monitor output was processed to provide minutes spent in MVPA to be equivalent to ≥2000 ActiGraph counts per minute;[21] further details on accelerometer data processing can be found elsewhere.[23]

2.4.6 Stage 4- Long-term compliance

Stage 4 used accelerometer measurements taken at 10-months post-intervention to reflect long-term compliance. Average daily minutes of MVPA was used as described above.

2.4.7 Stage 5- Evaluation participation

During stage 5 differential participation to evaluation measures was assessed as compliance to questionnaire and accelerometer measures. Questionnaire compliance was defined as whether participants provided questionnaire data at each measurement occasion. Research staff working on the GoActive study recorded whether a questionnaire for each participant had been completed and checked at each measurement point. Accelerometer compliance was determined as whether participants provided valid accelerometer data at each measurement point. In line with the main GoActive trial analysis, participants were required to provide 6 hours of wear time from a possible 42 hours in each day time quadrant: morning (06.00 to 12.00), afternoon (12.00 to 18.00), evening (20.00 to 24.00) and night (24.00 to 06.00).[23]

2.4.8 Stage 6- Health outcomes

Related health outcomes were explored during stage 6 using anthropometric measures. During a school site visit, trained measurement staff conducted the following measures according to standardised operation procedures: height (m), weight (kg), waist circumference (cm), and bio-impedance to assess body fat percentage (%).[21] BMI standard deviation scores (BMI SDS) were calculated from

height and weight data [i.e. weight/height2 (kg/m^2)] and categorised according to age and gender standardised International Obesity Task Force (IOTF) thresholds.[23]

2.9 Analysis

Characteristics of the sample were described using mean, standard deviation and frequency values. Data from all measurement points were included across the analyses described below and were stratified by either individual- or school-level SEP.

Research questions under stages 1 and 2 used self-reported data from the school environment and student questionnaires. Data were explored using simple tabulations, graphical techniques and basic summary statistics and analysed using Kruskal-Wallis one-way analysis of variance by school-level SEP. This test was selected due to the skewness of the data. P-values were adjusted for ties as the same values occurred in more than one sample. For further analyses under stage 2, website access by the intervention group was also explored using Pearson's chi-squared. Of those who accessed the website, differences in the number of visits and points logged by individual-level SEP were analysed using the Kruskal-Wallis test as described above.

Research questions under stages 3 and 4 were explored using accelerometer assessed MVPA, interaction analyses were run to examine if the effect of the independent variable (intervention vs control) on the dependent variable (daily average MVPA) differed by individual-level SEP, following statistical procedures from the main GoActive analyses.[21] For MVPA at T3 and T4 (i.e. the primary outcome), the intervention effect, representing the baseline-adjusted difference in change from baseline between the intervention and control groups, was estimated from a linear regression model, including randomisation group, baseline value of the outcome [i.e. analysis of covariance (ANCOVA)] the randomisation stratifiers (i.e. pupil premium funding and county), and an interaction between individual-level SEP and group allocation. Models were also run separately for low and middle/high socioeconomic groups to assess intervention effects within subgroups. Robust standard errors (SEs) were calculated to allow for the non-independence of individuals within schools.

For peer review only

Under stage 5, we examine differential response to evaluation measures by individual-level SEP. We examined accelerometer compliance and self-report compliance (e.g. questionnaire completion vs no completion) using Pearson's chi-squared.

Stage 6 was explored using anthropometric outcomes. Interaction analyses were used to examine if the effect of the independent variable (intervention vs control) on the dependent variable (BMI, waist circumference or body fat) differed by individual-level SEP, separate analyses were run for each anthropometric variable following the same analytical approach as stages 3 and 4.

All analyses were conducted using STATA V.15.1 software.

2.10 Patient and Public Involvement

None

For peer review only

3. Results

3.1 Sample description

A total of 2838 students provided baseline questionnaire data. Table 1 provides an overview of baseline characteristics by individual-level SEP. Overall, mean age was 13.3 (SD 0.4) years, just over half of participants were male (51.4%) and the majority of participants were of White British ethnicity (84.7%). Fewer participants were of a low-SEP (14.0%), than of middle- (42.5%) and high-SEP (43.5%).

Table 1: Baseline descriptive characteristics by individual-level SEP

	**Low-SEP**	**Middle-SEP**	**High-SEP**
	**N (%)**		
Participant	398 (14.0)	1206 (42.5)	1234 (43.5)
**Gender**			
Male	196 (6.9)	598 (21.1)	684 (24.1)
Female	202 (7.1)	608 (21.4)	550 (19.4)
**Ethnic group**			
White	319 (11.3)	1032 (36.5)	1071 (37.8)
Mixed/multiple ethnic background	32 (1.1)	73 (2.6)	76 (2.7)
Asian or Asian British	20 (0.7)	52 (1.8)	36 (1.3)
Black or black British	16 (0.6)	30 (1.1)	24 (0.8)
Other ethnic group	10 (0.4)	16 (0.6)	22 (0.7)
	**Mean (SD)**		
Age	13.2 (0.4)	13.3 (0.4)	13.3 (0.5)
BMI	21.1 (4.3)	20.5 (3.7)	20.1 (3.5)
Body fat %	22.1 (10.2)	21.3 (10.1)	19.9 (9.7)
Waist circumference	71.7 (11.1)	70.5 (9.7)	69.0 (8.9)

3.2 Main analyses

3.2.1 Stage 1-Provision and access

Table 2 shows that regardless of school-level SEP, teachers reported their schools to be suitable for physical activity at baseline. Differences between the provision of and access to physical activity facilities by school-level SEP were tested, but none were identified as statistically significant with p-values >0.05.

For peer review only

Table 2: Physical activity environment and recruitment by school-level SEP

	Schools of low-SEP	Schools of high-SEP
	**Mean (SD)**	**Mean (SD)**
**Physical Activity Environment**		
**School level measure (possible range)**		
Quality of school physical activity facilities (0-3)	2.6 (0.5)	2.5 (0.4)
Suitability of school grounds for physical activity (3-9)	8.3 (1.5)	8.0 (1.4)
Extra-curricular opportunities for physical activity (0-25)	11.0 (2.2)	12.5 (3.7)
Weekly hours of Physical Education (0+)	2.0 (0.0)	2.2 (.4)
Area around school suitable for physical activity (3-15)	11.9 (2.2)	12.8 (1.2)
School attitude towards physical activity (5-25)	18 (6.0)	19.3 (6.3)
**Recruitment rates**		
Number of Year 9 students at baseline (N)	1648	1759
Recruited at baseline N (%)	1369 (83.1)	1469 (83.5)
**Students from each family affluence group by school-level SEP**	**N (%)**	**N (%)**
Low individual-SEP	266 (19.4)	132 (9.0)
Middle individual-SEP	598 (43.7)	608 (41.4)
High individual-SEP	505 (36.9)	729 (49.6)

Note: Higher scores= more favourable facilities, opportunities, environment or attitude

3.2.2 Stage 2- Intervention uptake

Table 2 provides a breakdown recruitment by school-level SEP, suggesting that a lower proportion of students from low-SEP were recruited into the GoActive trial, particularly in high-SEP schools.

Table 3 presents the uptake of the GoActive intervention using website engagement. The results show that significantly fewer students of low-SEP than middle- and high-

For peer review only

SEP accessed the GoActive intervention website, but no difference in engagement for those that did access the website.

Table 3: Website access and engagement of students in the intervention group

	Low-SEP N=235	Middle-SEP N=670	High-SEP N=606	X^2	d.f	P-value (adjusted for ties)
**Accessed the website N (%)**	94 (40.0)	304 (45.4)	315 (52.0)	16.52	2	0.00
**Mean website points (SD)**	49.8 (123.1)	53.2 (85.1)	55.0(87.8)	0.53	2	0.77
**Mean website visits (SD)**	14.2 (28.1)	15.5 (21.0)	16.0 (22.8)	0.74	2	0.69

3.2.3 Stage -Intervention effect

Table 4 shows the moderating effect of SEP on the effectiveness of the GoActive intervention on average daily minutes of MVPA. The results of the interaction analysis suggest at the post-intervention the intervention effect in students of middle/high-SEP was 4.56 (95%CI: -9.56 to 0.41) minutes/day less MVPA than students of low-SEP. However, subgroup analyses did not show statistically significant effects in either group.

Table 4: Intervention effect on daily accelerometer assessed MVPA at 14-16 weeks and 10-months post intervention and on anthropometric measures at 10-months post intervention

	B	95% CI	P-value	Model N
**14-16 weeks post intervention**				
**MVPA**				
Interaction term				
Intervention*SEP	-4.56	-9.56 to 0.41	0.069	1878
Stratified analysis				
Low-SEP	3.13	-1.27 to 7.54	0.150	241
Middle/High-SEP	-1.49	-6.54 to 3.57	0.540	1637
**10-months post intervention**				
**MVPA**				

For peer review only

Interaction term				
Intervention*SEP	-7.53	-12.89 to -2.17	0.009	1785
Stratified analysis				
Low-SEP	4.90	0.09 to 9.70	0.046	203
Middle/High-SEP	-2.76	-6.78 to 1.26	0.164	1582
**BMI z-score**				
Interaction effect				
Intervention*SEP	0.12	-0.02 to 0.26	0.096	2070
Stratified analysis				
Low-SEP	-0.10	-0.19 to 0.0	0.055	247
Middle/High-SEP	0.03	-0.05 to 0.12	0.413	1823
**Body fat (%)**				
Interaction term				
Intervention*SEP	1.09	-0.63 to 2.81	0.198	1873
Stratified analysis				
Low-SEP	-0.69	-3.17 to 1.78	0.560	216
Middle/High-SEP	0.41	-0.75 to 1.57	0.464	1657
**Waist circumference (cm)**				
Interaction term				
Intervention*SEP	0.73	-0.68 to 2.15	0.287	2089
Stratified analysis				
Low-SEP	-0.71	-1.64 to 1.30	0.808	249
Middle/High-SEP	0.56	-0.17 to 1.30	0.124	1840

Note: All models adjusted for school-level SEP, county and school clustering; MVPA models also adjusted for baseline MVPA

3.2.4 Stage 4- Long-term compliance

At 10-months post intervention, the difference in intervention effect increased to 7.53 (95%CI: -12.89 to -2.17) minutes/day MVPA in favour of participants of low-SEP (Table 4). Subsequent stratified analyses showed a positive intervention effect in participants of a low-SEP (4.90; 0.09 to 9.70) but not those of middle/high.

3.2.5 Stage 5- Evaluation participation

Figure 3a shows that questionnaire compliance decreased throughout the intervention across all socioeconomic groups. The figure also shows an association between individual-level SEP and questionnaire compliance (lower compliance among students of low-SEP). The strength of this association increased from T2 to T3 to T4

For peer review only

(**T2:** X^2 = 23.45, *p* = 0.00; **T3:** X^2 = 15.25, *p* = 0.00; **T4:** X^2 = 43.88, *p* = 0.00). Figure 3b shows this trend was also observed for accelerometer compliance (**BL:** X^2 = 8.90, *p* = 0.02; **T3:** X^2 = 8.12, *p* = 0.02; **T4:** X^2 = 33.65, *p* = 0.00).

Insert figure 3

3.2.6 Stage 6-Health outcomes

Table 4 shows an indication (p=0.09) of a more favourable intervention effect for participants of low-SEP on BMI z-score, however this did not reach statistical significance. No interaction effects were observed for waist circumference or body fat. Subsequent stratified analyses suggest a favourable intervention effect on BMI z-score among adolescents of low-SEP when compared to the control condition (Low SEP: -0.10; 95% CI:-0.19,0.00), but not for those of middle-high-SEP (middle/high: 0.03; 95% CI:-0.05,0.12).

See supplementary file 1 for man physical activity and anthropometric outcomes by socioeconomic position and randomisation group at each measurement point.

For peer review only

4. Discussion

Taking a case-study approach we investigated if and how socioeconomic inequalities arose during the implementation and evaluation of a school-based physical activity intervention called GoActive. In doing so, we present a novel approach to analysing young people's physical activity intervention from an equality lens. Our findings demonstrate the benefit of taking this approach to intervention evaluation, providing insight beyond the main trial analysis.

We discuss our main findings in relation to three key elements: intervention context and engagement (stages 1 & 2), intervention effectiveness (stages 3, 4 & 6) and intervention recruitment and evaluation (stages 2 & 5).

4.1 Intervention context and engagement

Our finding that school-level SEP did not appear to influence the school physical activity environment at baseline, contrasts with previous research highlighting socioeconomic disparities in school physical activity provision and resources.[6,31,32] Whilst it is likely this could be the result of the small sample size of included schools (n=16) and resultant limited power to show significant differences, this could also be due to the UK context of GoActive, where national and local policy, such as the Schools Premises Regulations, impose minimum standards for school sports grounds and facilities.[33,34] In relation to engagement, our findings show that despite being exposed to similar school environments, significantly fewer adolescents of low-SEP accessed the GoActive website. Of those who did, a graded effect was observed with adolescents of low-SEP engaging the least. One explanation for this, as highlighted in previous research, is that students living in the context of socioeconomic deprivation interact differently with the school environment (e.g., the use of equipment, fostering of autonomy, competence and relatedness, update of extracurricular opportunities) potentially impacting their engagement with GoActive.[35,36] Furthermore, review evidence reports that adolescents of low-SEP experience multiple barriers to engaging in physical activity interventions, including digital exclusion.[12]

For peer review only

4.2 Intervention Effectiveness

Despite apparently lower engagement, our exploratory analyses suggest that participants of a low-SEP responded more favourable to GoActive, with a difference in effect of 7.53 minutes/day at 10-months post-intervention to participants of a middle/high-SEP. It may be that students of a low-SEP had a lower engagement with the website but were more engaged with other elements of the intervention that we have no data on. The observed intervention effect of ~5 minutes of MVPA per day may be important for health,[37] and was the targeted effect in main Goactive trial. A similar pattern of effect was also observed for BMI z-score.[21] Overall, these findings support the potential for school-based intervention to reduce inequalities in physical activity and obesity. It is possible more deprived students particularly benefited from the chance to try the variety of new activities offered during the GoActive intervention.[6,23] This is especially promising given the stark socioeconomically patterned inequalities in overweight and obesity in the UK and other high-income countries. [6,38]

Our choice to treat adolescents of low-SEP as an independent group was based on recent review evidence that their experiences of physical activity notably differ to those of middle- and high-SEP, highlighting the value of looking at them as a separate group.[6] By doing so, our findings add to the main trial moderation analyses where participants of low-and middle-SEP were grouped, suggesting the intervention effect was primarily experienced among low-SEP adolescents. While the approach initially taken was pre-specified and common among existing literature, mainly due to the small sample size of low-SEP groups, these exploratory analyses suggest that important differences in effect may be overlooked at as a result.

4.3 Intervention recruitment and evaluation

Recruitment data showed that 14% of those participating in the GoActive trial were of a low-SEP and the majority of these students attended low-SEP schools. In the East of England, data from the Family Resources Survey (2016-2019) reports 19.5% of young

For peer review only

people were living in poverty at the time GoActive was implemented.[39] It is possible this is due to the small sample of 16 schools, that are unlikely to be representative of the county. Furthermore, whilst ‘living in poverty’ is a different indicator of SEP than family affluence, measures of SEP are shown to be highly correlated.[24,40] It is therefore worth considering, when comparing these percentages that adolescents of low-SEP might be underrepresented in the overall GoActive sample, aligning with evidence that socioeconomically disadvantaged groups are “hard to reach” and recruit into research.[41] Of those recruited into GoActive, inequalities in study participation were observed. These results are consistent with previously reported socioeconomic patterns in response to survey evaluation measures.[42] [8,43] Higher accelerometer non-response has also been reported among socioeconomically deprived children,[44,45] however, there is a lack of research looking at socioeconomic patterning in accelerometer compliance among adolescent populations.

Based on these findings, it is important to acknowledge that our analyses were conducted using a small subset of students of low-SEP which may result in bias in our conclusions. It is possible that differential engagement and response to evaluation measures resulted in a subset of students of low-SEP who were not reflective of the group more broadly, impacting the generalisability of our results. Furthermore, it is possible this may have impacted the results of our analyses under stages 3 and 4, where those who remained in GoActive are more likely to be those who got most out of it.

4.4 Strengths and limitations

Previous research has begun to look at differential effectiveness using the primary outcome of the intervention.[7] To our knowledge, this is the first paper to provide an example of how inequalities can be explored throughout the intervention and research process of young people’s physical activity interventions. We demonstrate how taking this approach provides additional insight for the development of effective and equitable interventions. Further strengths include the diversity of data collected during the GoActive trial which allowed us to build a more holistic picture of

For peer review only

inequalities during the trial and the use of device-measured MVPA, which aligns with public health research recommendations for the objective and comprehensive evaluation of health promotion programmes.[23,46]

Whilst presenting our result as exploratory, rather than confirmatory, we acknowledge the small sample size of the low-SEP group and school-level data raises problems with regards to statistical power.[47] The subjective quantification of school environment features may have given rise to self-report biases.[35] It is possible that teachers' reported acceptability of physical activity resources was relative to school-level deprivation, with teachers at high-SEP schools expecting a higher standard of resources and facilities. It is also suggested that some activity types (e.g. biking, stair walking) and intensities can be misclassified by wrist-worm accelerometers.[48] If these behaviours are also socioeconomically patterned, this may have led to an under or overestimation of the different in effect between subgroups. Further limitations include the relative lack of participants of a low-SEP and of non-white ethnicity.[23]

4.5 Recommendation for future research and practice

This paper presents a case-study example of how to analyse young people's physical activity interventions with an inequalities lens. Drawing on the stages developed by Love (2019), we provide a framework for future studies to apply, adapt and develop.[20] Whilst the paper focuses on young people's physical activity interventions, the application of this approach more broadly is encouraged. The data required for such a comprehensive analysis should be considered during the design stage of future interventions and trials, this will help prevent the development and implementation of unequal interventions, making better use of public resources.[49] Mobilising this approach for existing strategies will further help develop our understanding of why current interventions appear to be ineffective in tackling physical inactivity during adolescence.[10]

The results of this stage-based analysis show the potential for universal school-based physical activity interventions to positively impact socioeconomically deprived

students, reducing inequalities. The results also demonstrate how intervention components that require individual agency, such as accessing the GoActive website, can exacerbate inequalities.[50,51] This should be considered in the development and implementation of school policy, especially in schools with a high proportion of students of a low-SEP. Due to the exploratory nature of this study, it would be beneficial for future research to further study the potential benefit of school-based physical activity interventions for students of low-SEP. It may be useful to explore the application of easily accessible interventions, such as the Daily Mile, to a secondary school setting.[52]

Recruiting and retaining participants of a low-SEP can be challenging, which means they are often underrepresented in research.[12] To increase the reach of interventions and to be able to conduct statistically powered subgroup analyses, the development of active and targeted recruitment of adolescents living in the context of socioeconomic deprivation is an important area for future research. Strategies are also needed to better engage these adolescents in the research process, for example involving them in the design and research process through patient and public involvement (PPI).[53]

5.Conclusion

This was an exploratory study exploring whether and how socioeconomic inequalities might arise throughout a school-based physical activity intervention. We demonstrate how the GoActive trial positively affected the physical activity and BMI of low-SEP students. However, differential engagement in the intervention and response to evaluation measures may have biased these conclusions. The continued development and evaluation of school-based interventions from an inequalities lens is essential as we move out of the Covid-19 pandemic, where disparities in school-based physical activity were exacerbated.[54]

For peer review only

Contributorship statement

OA conceived the study in collaboration with EvS and HF. KC led the GoActive trial and provide feedback on this secondary analysis of the GoActive data, as did PW.

Competing interest

None

Funding

Olivia Alliott is funded by the National Institute for Health and Care Research (NIHR) School for Public Health Research (SPHR), Grant Reference Number PD-SPH-2015. Hannah Fairbrother is funded through the NIHR School for Public Health Children, young people & families programme. The work of Esther van Sluijs is supported by the Medical Research Council (grant number MC_UU_00006/5). The GoActive trial was funded by the National Institute for Health Research (NIHR) Public Health Research Programme (https://www.nihr.ac.uk/explore-nihr/funding-programmes/public-healthresearch.htm; award number: 13/90/18) and undertaken under the auspices of the Centre for Diet and Activity Research (CEDAR), a UKCRC Public Health Research Centre of Excellence. The GoActive trial was also supported by NIHR Biomedical Research Centre Cambridge: Nutrition, Diet, and Lifestyle Research Theme (Grant IS-BRC-1215-20014) to KC, EvS. The funders had no role in the GoActive study design, data collection and analysis, decision to publish, or preparation of the manuscript. The views expressed in this article are those of the author(s) and the funders had no role in the design of the study, data analysis, data interpretation or in writing the manuscript.

Data sharing

The authors do not have the authority to share the data that support the findings of this study and the data are not openly available because of ethical and legal considerations. Non-identifiable data can be made available to bona-fide researchers on submission of a reasonable request to datasharing@mrc-epid.cam.ac.uk. The principles and processes for accessing and sharing data are outlined in the MRC Epidemiology Unit Data Access & Data Sharing Policy.

For peer review only

Ethical Approval Statement

Ethical approval for the GoActive trial was obtained from University of Cambridge Psychology Research Ethics Committee (PRE.2015.126). No additional approval was sought for this study as it was a secondary data analysis.

Figure Legends

Figure 1: Points in the intervention process where inequalities may be generated, figure developed by Love (2019).[20]

Figure 2: Intervention stages and accompanying research questions explored throughout this study [20]

Figure 3 a and b: Compliance to study evaluation measures throughout the GoActive trial by individual level-SEP, (a) percentage of student proving questionnaire data; (b) percentage of students providing accelerometer data.

For peer review only

References

1. Pratt M, Varela AR, Salvo D, Kohl III HW, Ding D. Attacking the pandemic of physical inactivity: what is holding us back? Vol 54: BMJ Publishing Group Ltd and British Association of Sport and Exercise Medicine; 2020:760-762.
2. Blair SN. Physical inactivity: the biggest public health problem of the 21st century. *British journal of sports medicine.* 2009;43(1):1-2.
3. Aberg MA, Pedersen NL, Toren K, et al. Cardiovascular fitness is associated with cognition in young adulthood. *Proc Natl Acad Sci U S A.* 2009;106(49):20906-20911.
4. Guthold R, Stevens GA, Riley LM, Bull FC. Global trends in insufficient physical activity among adolescents: a pooled analysis of 298 population-based surveys with 1.6 million participants. *The Lancet Child & adolescent health.* 2020;4(1):23-35.
5. Vander Ploeg KA, Maximova K, McGavock J, Davis W, Veugelers P. Do school-based physical activity interventions increase or reduce inequalities in health? *Soc Sci Med.* 2014;112:80-87.
6. Alliott O, Ryan M, Fairbrother H, Van Sluijs E. Do adolescents' experiences of the barriers to and facilitators of physical activity differ by socioeconomic position? A systematic review of qualitative evidence. *Obes Rev.* 2021.
7. Love RE, Adams J, van Sluijs EMF. Equity effects of children's physical activity interventions: a systematic scoping review. *The international journal of behavioral nutrition and physical activity.* 2017;14(1):134.
8. White M, Adams J, Heywood P. How and why do interventions that increase health overall widen inequalities within populations? *Health, inequality and society*. Bristol: Policy Press 2009.
9. Rose G. Sick individuals and sick populations. 1985. *International journal of epidemiology.* 1985;14(1):32-38.
10. Neil-Sztramko SE, Caldwell H, Dobbins M. School-based physical activity programs for promoting physical activity and fitness in children and adolescents aged 6 to 18. *Cochrane Database of Systematic Reviews.* 2021(9).

For peer review only

11. Venkatraman T, Honeyford K, Costelloe CE, et al. Sociodemographic profiles, educational attainment and physical activity associated with The Daily Mile™ registration in primary schools in England: a national cross-sectional linkage study. *Journal of epidemiology and community health.* 2021;75(2):137-144.

12. Craike M, Wiesner G, Hilland TA, Bengoechea EG. Interventions to improve physical activity among socioeconomically disadvantaged groups: an umbrella review. *Int J Behav Nutr Phys Act.* 2018;15(1):1-11.

13. Frohlich KL, Potvin L. Transcending the known in public health practice: the inequality paradox: the population approach and vulnerable populations. *American journal of public health.* 2008;98(2):216-221.

14. Fernandes M, Sturm R. Facility provision in elementary schools: correlates with physical education, recess, and obesity. *Preventive medicine.* 2010;50:S30-S35.

15. Sallis JF, Conway TL, Prochaska JJ, McKenzie TL, Marshall SJ, Brown M. The association of school environments with youth physical activity. *American journal of public health.* 2001;91(4):618.

16. Beauchamp A, Backholer K, Magliano D, Peeters A. The effect of obesity prevention interventions according to socioeconomic position: a systematic review. *Obes Rev.* 2014;15(7):541-554.

17. Rush E, Reed P, McLennan S, Coppinger T, Simmons D, Graham D. A school-based obesity control programme: Project Energize. Two-year outcomes. *British Journal of Nutrition.* 2012;107(4):581-587.

18. Williams NA, Coday M, Somes G, Tylavsky FA, Richey PA, Hare M. Risk factors for poor attendance in a family-based pediatric obesity intervention program for young children. *Journal of developmental and behavioral pediatrics: JDBP.* 2010;31(9):705.

19. Craig CL, Bauman A, Gauvin L, Robertson J, Murumets K. ParticipACTION: A mass media campaign targeting parents of inactive children; knowledge, saliency, and trialing behaviours. *Int J Behav Nutr Phys Act.* 2009;6(1):1-7.

20. Love R. *Inequalities in Children's Physical Activity and Interventions*: Centre for Diet and Physical Activity Research, MRC Epidemiology Unit, University of Cambridge2019.

For peer review only

21. Brown HE, Whittle F, Jong ST, Croxson C, Sharp SJ, Wilkinson P. A cluster randomised controlled trial to evaluate the effectiveness and cost-effectiveness of the GoActive intervention to increase physical activity among adolescents aged 13–14 years. 2017;7:e014419.

22. Department of Education GU. Guidance Using pupil premium: guidance for school leaders. Published 18 October 2019; Last updated 1 November 2021.

23. Corder KL, Brown HE, Croxson CH, et al. A school-based, peer-led programme to increase physical activity among 13-to 14-year-old adolescents: the GoActive cluster RCT. 2021.

24. Galobardes B, Lynch J, Smith GD. Measuring socioeconomic position in health research. *British medical bulletin.* 2007;81(1):21.

25. Pardo-Crespo MR, Narla NP, Williams AR, et al. Comparison of individual-level versus area-level socioeconomic measures in assessing health outcomes of children in Olmsted County, Minnesota. *Journal of epidemiology and community health.* 2013;67(4):305-310.

26. Johnson MRD, Bhopal RS, Ingleby JD, Gruer L, Petrova-Benedict RS. A glossary for the first World Congress on Migration, Ethnicity, Race and Health. 2019;172:85-88.

27. Harrison F, van Sluijs EM, Corder K, Jones A. School grounds and physical activity: Associations at secondary schools, and over the transition from primary to secondary schools. *Health & place.* 2016;39:34-42.

28. Rowlands AV, Olds TS, Hillsdon M, et al. Assessing sedentary behavior with the GENEActiv: introducing the sedentary sphere. 2014.

29. Phillips LR, Parfitt G, Rowlands AV. Calibration of the GENEA accelerometer for assessment of physical activity intensity in children. *Journal of science and medicine in sport.* 2013;16(2):124-128.

30. Schaefer CA, Nigg CR, Hill JO, Brink LA, Browning RC. Establishing and evaluating wrist cutpoints for the GENEActiv accelerometer in youth. *Medicine and science in sports and exercise.* 2014;46(4):826.

31. Hollis JL, Williams AJ, Sutherland R, et al. A systematic review and meta-analysis of moderate-to-vigorous physical activity levels in elementary school physical education lessons. *Preventive medicine.* 2016;86:34-54.

For peer review only

32. Carlson JA, Mignano AM, Norman GJ, et al. Socioeconomic disparities in elementary school practices and children's physical activity during school. *Am J Health Promot.* 2014;28(3_suppl):S47-S53.

33. GOV.UK. The School Premises (England) Regulations 2012. 2012; https://www.legislation.gov.uk/uksi/2012/1943/contents/made. Accessed 30th November 2021.

34. Department of Education GU. Area guidelines for mainstream schools. 2014; https://assets.publishing.service.gov.uk/government/uploads/system/uploads/attachment_data/file/905692/BB103_Area_Guidelines_for_Mainstream_Schools.pdf. Accessed 30 November 2021.

35. Foubister C, Van Sluijs EM, Vignoles A, et al. The school policy, social, and physical environment and change in adolescent physical activity: An exploratory analysis using the LASSO. *PloS one.* 2021;16(4):e0249328.

36. Moore GF, Littlecott HJ, Evans R, Murphy S, Hewitt G, Fletcher A. School composition, school culture and socioeconomic inequalities in young people's health: Multi-level analysis of the Health Behaviour in School-aged Children (HBSC) survey in Wales. *British Educational Research Journal.* 2017;43(2):310-329.

37. Ekelund U, Luan J, Sherar LB, Esliger DW, Griew P, Cooper A. Moderate to vigorous physical activity and sedentary time and cardiometabolic risk factors in children and adolescents. *Jama.* 2012;307(7):704-712.

38. Mayor S. Socioeconomic disadvantage is linked to obesity across generations, UK study finds. British Medical Journal Publishing Group; 2017.

39. Agrawal S, Phillips D. Catching up or falling behind? Geographical inequalities in the UK and how they have changed in recent years. *The Institute for Fiscal Studies.* 2020:p2.

40. Galobardes B, Shaw M, Lawlor DA, Lynch JW, Davey Smith G. Indicators of socioeconomic position (part 1). *Journal of epidemiology and community health.* 2006;60(1):7-12.

41. Bonevski B, Randell M, Paul C, et al. Reaching the hard-to-reach: a systematic review of strategies for improving health and medical research with socially disadvantaged groups. *BMC medical research methodology.* 2014;14(1):1-29.

For peer review only

42. Turrell G, Patterson C, Oldenburg B, Gould T, Roy M-A. The socio-economic patterning of survey participation and non-response error in a multilevel study of food purchasing behaviour: area-and individual-level characteristics. *Public Health Nutr.* 2003;6(2):181-189.

43. Ligthart KA, Buitendijk L, Koes BW, van Middelkoop M. The association between ethnicity, socioeconomic status and compliance to pediatric weight-management interventions–a systematic review. *Obes Res Clin Pract.* 2017;11(5):1-51.

44. Rich C, Cortina-Borja M, Dezateux C, et al. Predictors of non-response in a UK-wide cohort study of children's accelerometer-determined physical activity using postal methods. *BMJ open.* 2013;3(3):14.

45. Love R, Adams J. Socioeconomic and ethnic differences in children's vigorous intensity physical activity: a cross-sectional analysis of the UK Millennium Cohort Study. 2019;9(5):e027627.

46. All-Party Commission on Physical A. Tackling Physical Inactivity – A Coordinated Approach. 2014.

47. Haas JP. Sample size and power. *American journal of infection control.* 2012;40(8):766-767.

48. Arvidsson D, Fridolfsson J, Borjesson M. Measurement of physical activity in clinical practice using accelerometers. *Journal of internal medicine.* 2019;286(2):137-153.

49. Wight D, Wimbush E, Jepson R, Doi L. Six steps in quality intervention development (6SQuID). *Journal of epidemiology and community health.* 2016;70(5):520-525.

50. Coggon J, Adams J. 'Let them choose not to eat cake...': Public health ethics, effectiveness and equity in government obesity strategy. *Future Healthcare Journal.* 2021;8(1):49.

51. Adams J, Mytton O, White M, Monsivais P. Why are some population interventions for diet and obesity more equitable and effective than others? The role of individual agency. *PLoS medicine.* 2016;13(4):e1001990.

For peer review only

52. Marchant E, Todd C, Stratton G, Brophy S. The Daily Mile: Whole-school recommendations for implementation and sustainability. A mixed-methods study. *PloS one.* 2020;15(2):28.

53. McDonagh JE, Bateman B. 'Nothing about us without us': considerations for research involving young people. *Archives of Disease in Childhood-Education and Practice.* 2012;97(2):55-60.

54. Ng K, Cooper J, McHale F, Clifford J, Woods C. Barriers and facilitators to changes in adolescent physical activity during COVID-19. *BMJ open sport & exercise medicine.* 2020;6(1):e000919.

For peer review only

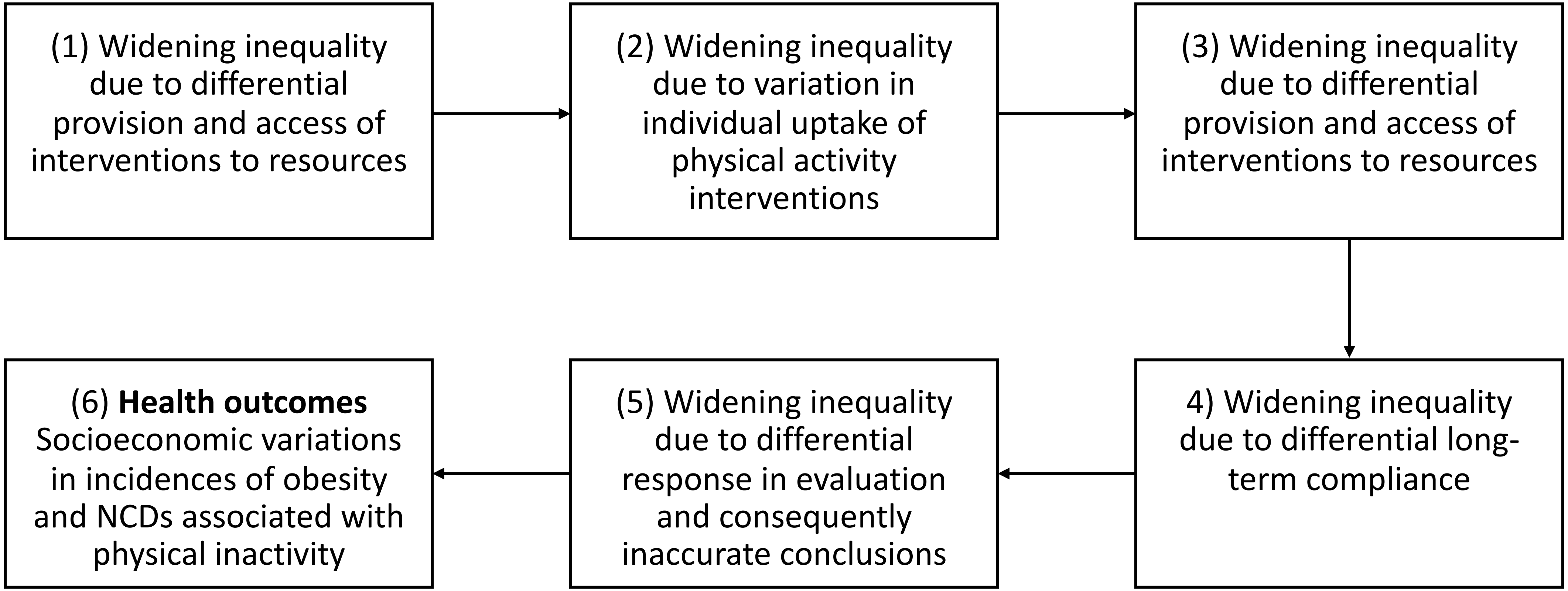

For peer review only - http://bmjopen.bmj.com/site/about/guidelines.xhtml

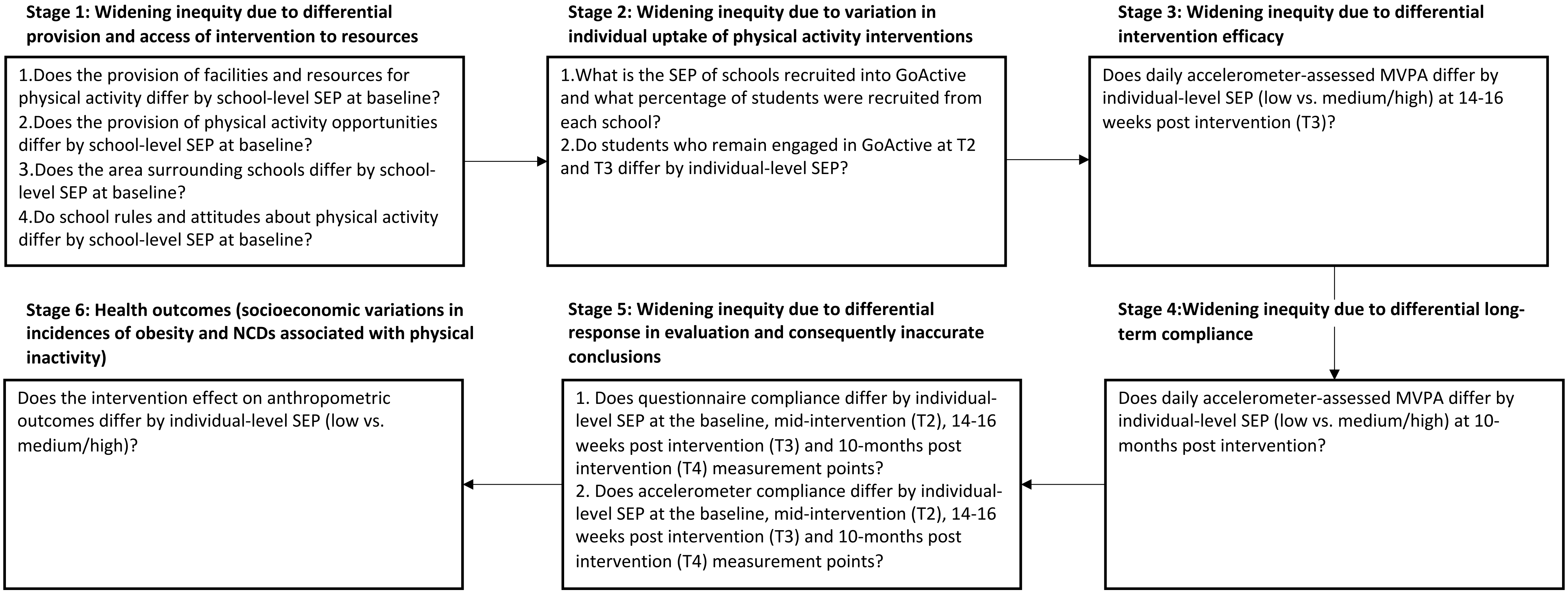
Stage 1: Widening inequity due to differential provision and access of intervention to resources
1.Does the provision of facilities and resources for physical activity differ by school-level SEP at baseline?
2.Does the provision of physical activity opportunities differ by school-level SEP at baseline?
3.Does the area surrounding schools differ by school-level SEP at baseline?
4.Do school rules and attitudes about physical activity differ by school-level SEP at baseline?
Stage 2: Widening inequity due to variation in individual uptake of physical activity interventions
1.What is the SEP of schools recruited into GoActive and what percentage of students were recruited from each school?
2.Do students who remain engaged in GoActive at T2 and T3 differ by individual-level SEP?
Stage 3: Widening inequity due to differential intervention efficacy
Does daily accelerometer-assessed MVPA differ by individual-level SEP (low vs. medium/high) at 14-16 weeks post intervention (T3)?
Stage 4:Widening inequity due to differential long-term compliance
Does daily accelerometer-assessed MVPA differ by individual-level SEP (low vs. medium/high) at 10-months post intervention?
Stage 5: Widening inequity due to differential response in evaluation and consequently inaccurate conclusions
1. Does questionnaire compliance differ by individual-level SEP at the baseline, mid-intervention (T2), 14-16 weeks post intervention (T3) and 10-months post intervention (T4) measurement points?
2. Does accelerometer compliance differ by individual-level SEP at the baseline, mid-intervention (T2), 14-16 weeks post intervention (T3) and 10-months post intervention (T4) measurement points?
Stage 6: Health outcomes (socioeconomic variations in incidences of obesity and NCDs associated with physical inactivity)
Does the intervention effect on anthropometric outcomes differ by individual-level SEP (low vs. medium/high)?

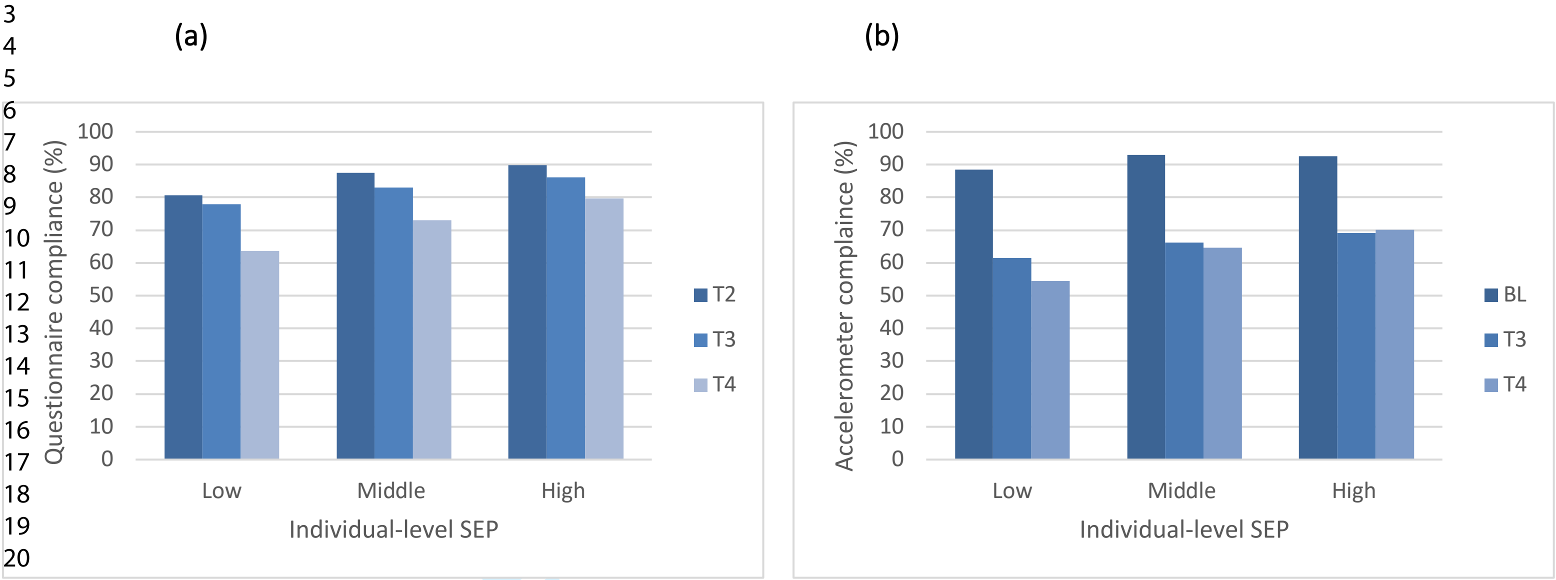
(a)
Questionnaire compliance (%)
100
90
80
70
60
50
40
30
20
10
0
Low
Middle
High
Individual-level SEP
T2
T3
T4
(b)
Accelerometer complaince (%)
100
90
80
70
60
50
40
30
20
10
0
Low
Middle
High
Individual-level SEP
BL
T3
T4

Table 1: Mean physical activity and anthropometric outcomes by socioeconomic position and randomisation group at each measurement point

	**Baseline**		**14-16 weeks post intervention**		**10-months post intervention**	
	Intervention	Control	Intervention	Control	Intervention	Control
**Low-SEP** *Mean (SD)*						
MVPA (minutes)	34.1 (15.4)	36.0 (20.9)	35.6 (21.0)	36.2 (21.7)	29.7 (24.0)	27.4 (17.5)
BMI z-Score	0.26 (2.06)	0.41 (1.71)			0.32 (1.93)	0.64 (1.33)
Body fat (%)	22.0 (9.6)	22.3 (11.1)			18.5 (12.8)	20.6 (12.9)
Waist circumference (cm)	71.2 (10.2)	72.5 (12.5)			72.4 (14.5)	74.8 (12.3)
**Middle/high-SEP** *Mean (SD)*						
MVPA (minutes)	35.9 (18.7)	35.5 (18.7)	33.3 (22.3)	35.4 (21.4)	25.0 (21.0)	27.7 (20.9)
BMI z-Score	0.10 (1.90)	0.17 (1.63)			0.10 (1.88)	0.15 (1.76)
Body fat (%)	20.7 (9.9)	20.5 (9.9)			18.2 (11.7)	18.6 (11.5)
Waist circumference (cm)	70.2 (9.5)	69.7 (9.1)			70.4 (14.3)	70.7 (13.1)

For peer review only

BMJ Open

Do socioeconomic inequities arise during school-based physical activity interventions? An exploratory case study of the GoActive trial

Journal:	*BMJ Open*
Manuscript ID	bmjopen-2022-065953.R1
Article Type:	Original research
Date Submitted by the Author:	20-Feb-2023
Complete List of Authors:	Alliott, Olivia; University of Cambridge, MRC Epidemiology Unit<br>Fairbrother , Hannah; University of Sheffield, Health Sciences School<br>Corder, Kirsten ; University of Cambridge, MRC Epidemiology Unit<br>Wilkinson, Paul; University of Cambridge, Cambridge Neuroscience<br>van Sluijs, Esther; University of Cambridge, MRC Epidemiology Unit
**Primary Subject Heading**:	Public health
Secondary Subject Heading:	Paediatrics
Keywords:	EPIDEMIOLOGY, PUBLIC HEALTH, Community child health < PAEDIATRICS

SCHOLARONE™
Manuscripts

I, the Submitting Author has the right to grant and does grant on behalf of all authors of the Work (as defined in the below author licence), an exclusive licence and/or a non-exclusive licence for contributions from authors who are: i) UK Crown employees; ii) where BMJ has agreed a CC-BY licence shall apply, and/or iii) in accordance with the terms applicable for US Federal Government officers or employees acting as part of their official duties; on a worldwide, perpetual, irrevocable, royalty-free basis to BMJ Publishing Group Ltd ("BMJ") its licensees and where the relevant Journal is co-owned by BMJ to the co-owners of the Journal, to publish the Work in this journal and any other BMJ products and to exploit all rights, as set out in our licence.

The Submitting Author accepts and understands that any supply made under these terms is made by BMJ to the Submitting Author unless you are acting as an employee on behalf of your employer or a postgraduate student of an affiliated institution which is paying any applicable article publishing charge ("APC") for Open Access articles. Where the Submitting Author wishes to make the Work available on an Open Access basis (and intends to pay the relevant APC), the terms of reuse of such Open Access shall be governed by a Creative Commons licence – details of these licences and which Creative Commons licence will apply to this Work are set out in our licence referred to above.

Other than as permitted in any relevant BMJ Author's Self Archiving Policies, I confirm this Work has not been accepted for publication elsewhere, is not being considered for publication elsewhere and does not duplicate material already published. I confirm all authors consent to publication of this Work and authorise the granting of this licence.

For peer review only

Do socioeconomic inequities arise during school-based physical activity interventions? An exploratory case study of the GoActive trial

Olivia Alliott[1], Hannah Fairbrother[2], Kirsten Corder[1], Paul Wilkinson[3] & Esther van Sluijs[1]

1. MRC Epidemiology Unit, University of Cambridge School of Clinical Medicine, Cambridge, UK
2. Health Sciences School, University of Sheffield, Sheffield, UK
3. Department of Psychiatry, University of Cambridge, Cambridge, UK

Correspondence to:

Dr Esther van Sluijs

MRC Epidemiology Unit, University of Cambridge School of Clinical Medicine, Cambridge, UK

Email: esther.vansluijs@mrc-epid.cam.ac.uk

For peer review only

Abstract

Objective To investigate socioeconomic inequities in the intervention and evaluation process of the GoActive school-based physical activity intervention and demonstrate a novel approach to evaluating intervention-related inequalities.

Design Exploratory post-hoc secondary data analysis of trial data.

Setting The GoActive trial was run in secondary schools across Cambridgeshire and Essex (UK), between September 2016 and July 2018.

Participants 13-14-year-old adolescents (n=2838, 16 schools).

Methods Socioeconomic inequities across 6 stages in the intervention and evaluation process were evaluated: (1) provision of and access to resources; (2) intervention uptake; (3) intervention effectiveness (accelerometer-assessed moderate-to-vigorous physical activity (MVPA)); (4) long-term compliance; (5) response in evaluation; and (6) impact on health. Data from self-report and objective measures were analysed by individual- and school-level socioeconomic position (SEP) using a combination of classical hypothesis tests and multilevel regression modelling.

Results Stage: (1) There was no difference in the provision of physical activity resources by school-level SEP (e.g. quality of facilities (0-3), low=2.6 (0.5); high=2.5 (0.4). (2) Students of low-SEP engaged significantly less with the intervention (e.g. website access: low=37.2%; middle=45.4%; high=47.0%; p=0.001). (3) There was a positive intervention effect on MVPA in adolescents of low-SEP (3.13 minutes/day, 95% CI -1.27, 7.54, but not middle/high (-1.49;-6.54, 3.57). (4) At 10-months post-intervention, this difference increased (low SEP: 4.90; 0.09, 9.70; middle/high SEP: -2.76; -6.78, 1.26). (5) There was greater non-compliance to evaluation measures among adolescents of low-SEP (e.g. % accelerometer compliance (low vs. high): baseline: 88.4 vs. 92.5; post-intervention: 61.6 vs. 69.2; follow-up: 54.5 vs. 70.2; p=0.00). (6) The intervention effect on BMI z-score was more favourable in adolescents of low-SEP (low SEP: -0.10; -0.19, 0.00; middle/high: 0.03; -0.047, 0.12).

For peer review only - http://bmjopen.bmj.com/site/about/guidelines.xhtml

Conclusions These analyses suggest the GoActive intervention had a more favourable positive effect on MVPA and BMI in adolescents of low-SEP, despite lower intervention engagement. However, differential response to evaluation measures may have biased these conclusions. We demonstrate a novel way of evaluating inequities within young people's physical activity intervention evaluations.

Strengths and limitations of this study

- We present a novel approach to evaluating inequities throughout the intervention process of young people's physical activity interventions.
- Whilst exploratory rather than confirmatory, the small sample size of the low-SEP group raises problems with regard to statistical power.
- The conclusions may be affected by attrition bias, as attrition was largest among participants of a low-SEP.

For peer review only - http://bmjopen.bmj.com/site/about/guidelines.xhtml

Introduction

The health benefits of physical activity are well-established[1] and physical inactivity has been identified as a major public health concern.[2] Active adolescents experience better present and long term health and are more likely to become active and healthy adults.[3-5] However, globally over 80% of students aged 11-17 years are insufficiently active to accrue the benefits.[6]

Similar to other health behaviours, disparities in physical activity during adolescence may contribute to inequities in current and future health.[7] Recent review-level evidence highlights the importance of promoting and enabling physical activity among adolescents living in the context of socioeconomic deprivation, who report experiencing more barriers to physical activity when compared to other socioeconomic groups.[8] Despite regularly collecting relevant information at baseline, most controlled trials of physical activity interventions in young people do not analyse differences in intervention effect across socioeconomic groups.[9] This has led to a scarcity of evidence regarding the differential impact of intervention across socioeconomic groups.[9]

Public health literature suggests the extent to which inequities are perpetuated or reduced can depend on the nature of the intervention.[10] ‘High-risk strategies’ target individuals with a higher risk of developing the disease, whereas population strategies attempt to lower the risk of the entire population by shifting the distribution of underlying risk factors, such as physical inactivity.[11] As a consequence of compulsory education in many countries, the potential for schools to deliver wide-reaching and equitable physical activity interventions has been well documented.[12,13] Taking a population approach, school-based interventions have been studied and deemed successful if average physical activity levels increase.[14] However, population strategies have the potential to inadvertently exacerbate health inequities within a population.[15]

Researchers have begun to consider the potential for interventions to have a differential effect across individuals, commonly named ‘intervention generated inequities’[10] However, across young people’s physical activity literature these studies have tended to focus on

For peer review only - http://bmjopen.bmj.com/site/about/guidelines.xhtml

differential effects by gender.[9] Limited evidence from individual evaluations of school-based interventions and physical activity interventions document socioeconomic inequities negatively impacting those of a low-SEP in the provision of, and access to, interventions and resources,[16,17] variation in intervention uptake,[18] differential intervention efficacy,[17,19] differential long-term compliance[20] and differential response in evaluations.[9,21,22]

These previous studies offer examples of various points in the research and intervention process where inequities might emerge. Going forward we propose a broader approach is needed, looking at intervention generated inequities throughout the whole research and intervention process of a single intervention. Based on the work of White et al., (2009),[10] Love (2019) identifies key stages throughout the physical activity intervention where inequities can be introduced.[22] Understanding how inequities might emerge at each of these stages is essential for the development of equitable school-based physical activity interventions, as whilst inequities at each of these stages could be small, together they may lead to significant inequities in final outcomes.[10]

The aim of this paper is to take a case-study approach to investigate if and how socioeconomic inequities arise during the intervention and evaluation process of the GoActive school-based physical activity intervention. In doing so, we demonstrate a novel way of studying inequities across the intervention implementation and evaluation process that could be applied more broadly.

Methods

This paper describes exploratory secondary analyses of the GoActive trial data. These analyses were not detailed in the Statistical Analysis Plan for the main trial analyses, but were guided by a pre-specific statistical analysis plan. The GoActive trial was run between September 2016 and July 2018. Ethical approval for the GoActive trial was obtained from the University of Cambridge Psychology Research Ethics Committee (PRE.2015.126). The trial was prospectively registered (ISRCTN31583496).

Participants and randomisation

For peer review only - http://bmjopen.bmj.com/site/about/guidelines.xhtml

Sixteen state-run secondary schools in Cambridgeshire and Essex agreed to participate. All Year 9 students (age 13-14-years) and their parents/carers received written study information. Students provided written assent and parents provided passive informed consent (opt-out consent).[23] School-level randomisation, stratified by the percentage of students eligible for Pupil Premium funding at each school (below or above county-specific median) and county (Cambridgeshire or Essex), occurred after baseline measurement.[23] Pupil premium funding aims to reduce the effects of deprivation on educational attainment and is used here as a proxy measure for school-level deprivation.[24]

GoActive intervention

GoActive was a theory-based intervention developed following an evidence-based iterative approach.[23] The primary aim of GoActive was to increase students' moderate-to-vigorous intensity physical activity (MVPA) across the week.[23] GoActive was delivered over 12 weeks to all students in the intervention schools irrespective of whether they participated in study measurements. The control schools followed normal practice.

GoActive was implemented using a tiered-leadership system led by mentors (older students within the school) and supported by peer-elected Year 9 leaders.[23] During the intervention, Year 9 tutor groups chose two activities per week from a selection of twenty. These activities required little or no equipment and were designed to appeal to a variety of students (including Ultimate Frisbee, Zumba and Hula Hoop). Schools had access to the GoActive intervention website where they could find activity instructions cards which included an overview of each activity, suggested adaptations, safety tips, 'factoids' and a short video.[23] Mentors remained with the class throughout the intervention, whereas peer-leaders changed each week. During the first six weeks, additional leadership was provided by a local authority-funded intervention facilitator (health trainers employed by local councils) who continued to provide remote support thereafter.[23]

Teachers were encouraged to dedicate one tutor time per week to do one of the chosen activities as a class. Students could gain points for trying these new activities at any time in or

For peer review only - http://bmjopen.bmj.com/site/about/guidelines.xhtml

out of school, irrespective of intensity or duration.[23] There was no expectation of time spent in the activities, points were rewarded for taking part. Individual points remained private and students could enter their points at any time on the GoActive website with an individual password and login details. Students were encouraged to regularly log these points to unlock rewards such as a sports bag, t-shirt, or hoodie. Whilst remaining private these points were entered into between-class competitions.[23]

The results of the main GoActive trail analysis reported no overall intervention effect on average daily MVPA.[25] Subgroup analyses conducted as part of the trial evaluation reported a suggestion of a positive intervention effect among students of a low/middle-SEP. Across all MVPA outcomes, those of high-SEP appeared to benefit least when compared to low/middle-SEP students. Full details of the trial methods have been published elsewhere.[23]

Methodological approach of the current study

As outlined above, we take a case study approach to demonstrate how socioeconomic inequities can be explored throughout the research and intervention process, using the GoActive intervention as an example. As this is an exploratory post-hoc analysis, we operationalised the model proposed by Love (2019) to include research questions based on the available GoActive data collected as part of the main GoActive trial (Figure 1).[22] For the remainder of this paper, we refer to the stages outlined in Figure 1 when describing our research and findings.

This paper focuses on socioeconomic inequities, therefore all of the research questions presented in Figure 1 consider SEP. We use individual- and area-level SEP, as using these different levels are important when evaluating the full contribution of socioeconomic conditions.[26] The relevance of different indicators of SEP is dependent upon on the research focus, health outcome and stage in the life course.[26] Taking this approach, we used pupil premium funding (see description in section 2.1) as a school-level indicator of SEP during stages 1 and 2, where the object of analysis is the school, not the individual. Schools were categories as low-SEP if the percentage of students eligible for Pupil Premium was below the county-specific mean and high-SEP if the percentage was above. For the remaining stages, we

For peer review only - http://bmjopen.bmj.com/site/about/guidelines.xhtml

use an individual-level indicator of SEP derived from the Family Affluence Scale (FAS).[27] In response to the recent review evidence highlighted in the introduction[8] (published after the main GoActive trial) and because of our focus on socioeconomic inequities we compare students of low-SEP to students of middle/high-SEP during stages 3, 4 and 5. This is a different approach to that of the main trail which grouped students of low- and middle-SEP together. All measures are described in further detail below.

Measures

Study measurements were taken at four time points during the GoActive trial: Baseline (BL), mid-intervention (T2; 6 weeks after intervention start, post-intervention (T3; 14–16 weeks after intervention start), and 10-month follow-up (T4; 10 months after intervention end.[25]

A summary of demographic measures and the measures specific to each stage are described below, the best available measures from the trial data were used to address the research questions under each stage. For conciseness, the following shorter titles are applied to each stage: stage 1 – provision and access, stage 2 - intervention uptake, stage 3 – intervention effect, stage 4 – long-term compliance, stage 5 – evaluation participation, stage 6 – health outcomes.

Demographic measures

Participant descriptive characteristics were self-reported at baseline.[23] Participants reported gender from 3 response options (male, female, prefer not to say).[25] Individual-level SEP was reported using the FAS, which is composed of six items relating to: (1) family car ownership, (2) holidays, (3) computers, (4) availability of bathrooms, (5) dishwasher ownership, and (6) having their own bedroom. These were used as a proxy measure of individual-level socioeconomic position by summing the answers (possible range 0–13), and dividing into predefined affluence groups (low = 0–6, middle = 7–9, high = 10–13).[25] Ethnicity was self-reported by participants, who were given 20 response options and an additional free text option.[25] The reported options were recoded into five categories in accordance with

For peer review only - http://bmjopen.bmj.com/site/about/guidelines.xhtml

published recommendations: (1) 'white', (2) 'mixed ethnicity' (i.e. identifying with multiple ethnicities), (3) 'Asian' (including South Asian and Chinese), (4) 'African and/or Caribbean' and (5) 'other'.[25,28]

Stage 1: Provision and access

Data on the school physical activity policy and social and physical environment were self-reported at baseline by contact teachers (often Physical Education or Year 9 lead) at all schools.[23]

The data were used to highlight the potential for socioeconomic differences in the provision of physical activity opportunities and access to resources at baseline, which may have impacted the delivery of GoActive. These data were collected using a questionnaire previously used in the Year 9 data collection of the Sport Physical Activity and Eating Behaviour, Environmental Determinants in Young People (SPEEDY) study.[29] A list of 16 physical activity facilities available at each school were given a quality rating (0=facility not present, 1= low quality facility, 2=middle quality facility, and 3=high quality facility). Ratings were summed and divided by the number of available facilities to give an average quality rating. An average rating was also used to indicate the suitability of the school grounds for sport, informal games and general play across three measures (1=not at all suitable, 2=somewhat suitable and 3=very suitable). The provision of physical activity opportunities was assessed using the extracurricular opportunities on offer at each school derived from a list of 24 (including space to add 'other' activities; one activity = one point e.g. Rounders = 1) and weekly hours of PE, measured using an open-ended question where teachers rounded to the nearest half-hour. The suitability of the area around the school for physical activity was assessed on a scale of 1-5 (1=strongly disagree to 5=strongly agree) across three measures, shielding from hedges/tress/fences, maintenance of the grounds and the presence of vandalism. Finally, the school's attitude towards physical activity was assessed using the same 1-5 agreement scale across five measures which included encouraging physical activity at school and outside school, educating about the risks of physical activity and how to practice safe physical activity and encouraging active travel.

For peer review only - http://bmjopen.bmj.com/site/about/guidelines.xhtml

Pupil premium was used as a school-level indicator of SEP, which was reported by teachers in the school environment questionnaire.[25]

Stage 2: Intervention uptake

Under stage 2, research questions explore engagement with the GoActive intervention. Recruitment data were used to assess the initial uptake of the intervention by school-level SEP. Evaluation uptake was measured as whether participants provided baseline questionnaire data, which was a requirement for participating in GoActive.[25] Trained measurement staff checked the questionnaires upon completion and helped students complete missing sections.[23] Intervention uptake was assessed using data on students' engagement with the GoActive website as this was the primary method for tracking the activities participants engaged in both in and out of school. This included whether students accessed the GoActive website at any time during the intervention period and was recorded as a categorical variable (accessed vs not). Of the students who did access the website, the number of times they visited and the number of points they logged throughout the intervention were recorded.

Stage 3: Intervention effect

During stage 3, differential intervention efficacy was explored for the GoActive primary outcome, daily accelerometer assessed MVPA at 14-16 weeks post-intervention.[23] Participants were asked to wear a wrist-worn activity monitor (Axivity) assessing acceleration (continuous waveform data) continuously (24 hours a day) for 7 days.[23] The axivity monitor has been validated to assess energy expenditure and to have increased wear time adherence and acceptability than hip-worn monitors in adolescents.[23,30-32] Monitor output was processed to provide minutes spent in MVPA to be equivalent to ≥2000 ActiGraph counts per minute;[23] further details on accelerometer data processing can be found elsewhere.[25]

Stage 4: Long-term compliance

For peer review only

Stage 4 used accelerometer measurements taken at 10-months post-intervention to reflect long-term compliance to the intervention by exploring compliance to the primary outcome after the intervention period. Average daily minutes of MVPA was used as described above.

Stage 5: Evaluation participation

During stage 5, differential participation in evaluation measures was assessed using compliance with questionnaire and accelerometer measures. Questionnaire compliance was defined as whether participants provided questionnaire data at each measurement occasion. Research staff working on the GoActive study recorded whether a questionnaire for each participant had been completed and checked at each measurement point. Accelerometer compliance was determined as whether participants provided valid accelerometer data at each measurement point. In line with the main GoActive trial analysis, participants were required to provide 6 hours of wear time from a possible 42 hours in each day time quadrant: morning (06.00 to 12.00), afternoon (12.00 to 18.00), evening (20.00 to 24.00) and night (24.00 to 06.00).[25]

Stage 6: Health outcomes

Related health outcomes were explored during stage 6 using anthropometric measures. During a school site visit, trained measurement staff conducted the following measures according to standardised operation procedures: height (m), weight (kg), waist circumference (cm), and bio-impedance to assess body fat percentage (%).[23] BMI standard deviation scores (BMI SDS) were calculated from height and weight data [i.e. weight/height2 (kg/m^2)] and categorised according to age and gender standardised International Obesity Task Force (IOTF) thresholds.[25]

Analysis

Characteristics of the sample were described using mean, standard deviation and frequency values. Data from all measurement points were included across the analyses described below

For peer review only - http://bmjopen.bmj.com/site/about/guidelines.xhtml

and were stratified by either individual- or school-level SEP. All included analyses were exploratory, but guided by an analysis plan developed prior to release of the data.

Research questions under stages 1 (provision and access) and 2 (intervention uptake) used self-reported data from the school environment and student questionnaires. Data were explored using simple tabulations, graphical techniques and basic summary statistics and analysed using Kruskal-Wallis one-way analysis of variance by school-level SEP. This test was selected due to the skewness of the data. P-values were adjusted for ties as the same values occurred in more than one sample. For further analyses under stage 2, website access by the intervention group was also explored using Pearson's chi-squared. Of those who accessed the website, differences in the number of visits and points logged by individual-level SEP were analysed using the Kruskal-Wallis test as described above.

Research questions under stages 3 (intervention effect) and 4 (long-term compliance) were explored using accelerometer assessed MVPA, interaction analyses were run to examine if the effect of the independent variable (intervention vs control) on the dependent variable (daily average MVPA) differed by individual-level SEP, following statistical procedures from the main GoActive analyses.[23] For MVPA at T3 and T4 (i.e. the primary outcome), the intervention effect, representing the baseline-adjusted difference in change from baseline between the intervention and control groups, was estimated from a linear regression model, including randomisation group, baseline value of the outcome [i.e. analysis of covariance (ANCOVA)] the randomisation stratifiers (i.e. pupil premium funding and county), and an interaction between individual-level SEP and group allocation. Models were also run separately for low and middle/high socioeconomic groups to assess intervention effects within subgroups. Robust standard errors (SEs) were calculated to allow for the non-independence of individuals within schools.

Under stage 5 (evaluation participation), we examine differential response to evaluation measures by individual-level SEP. We examined accelerometer compliance and self-report compliance (e.g. questionnaire completion vs no completion) using Pearson's chi-squared.

For peer review only - http://bmjopen.bmj.com/site/about/guidelines.xhtml

Stage 6 (health outcomes) was explored using anthropometric outcomes. Interaction analyses were used to examine if the effect of the independent variable (intervention vs control) on the dependent variable (BMI, waist circumference or body fat) differed by individual-level SEP, separate analyses were run for each anthropometric variable following the same analytical approach as stages 3 and 4.

All analyses were conducted using STATA V.15.1 software.

Patient and public involvement

None for the purpose of this secondary data analysis.

Results

Sample description

A total of 2838 students provided baseline questionnaire data. Table 1 provides an overview of baseline characteristics by individual-level SEP. Overall, mean age was 13.3 (SD 0.4) years, just over half of participants were male (51.4%) and the majority of participants were of White British ethnicity (84.7%). Fewer participants were of a low-SEP (14.0%), than of middle- (42.5%) and high-SEP (43.5%).

Table 1. Baseline descriptive characteristics by individual-level SEP

	**Low-SEP**	**Middle-SEP**	**High-SEP**
		**N (%)**	
Participant	398 (14.0)	1206 (42.5)	1234 (43.5)
**Gender**			
Male	196 (6.9)	598 (21.1)	684 (24.1)
Female	202 (7.1)	608 (21.4)	550 (19.4)
**Ethnic group**			
White	319 (11.3)	1032 (36.5)	1071 (37.8)
Mixed/multiple ethnic background	32 (1.1)	73 (2.6)	76 (2.7)
Asian or Asian British	20 (0.7)	52 (1.8)	36 (1.3)
Black or black British	16 (0.6)	30 (1.1)	24 (0.8)
Other ethnic group	10 (0.4)	16 (0.6)	22 (0.7)

For peer review only - http://bmjopen.bmj.com/site/about/guidelines.xhtml

Mean (SD)			
Age	13.2 (0.4)	13.3 (0.4)	13.3 (0.5)
BMI	21.1 (4.3)	20.5 (3.7)	20.1 (3.5)
Body fat %	22.1 (10.2)	21.3 (10.1)	19.9 (9.7)
Waist circumference	71.7 (11.1)	70.5 (9.7)	69.0 (8.9)

SEP=Socioeconomic position, SD=Standard deviation, BMI=Body mass index

Main analyses

Stage 1: Provision and access

Table 2 shows that regardless of school-level SEP, teachers reported their schools to be suitable for physical activity at baseline. Differences between the provision of and access to physical activity facilities by school-level SEP were tested, but none were identified as statistically significant with p-values >0.05.

Table 2. Physical activity environment and recruitment by school-level SEP

	**Schools of low-SEP (N=8)**	**Schools of high-SEP (N=8)**
	**Mean (SD)**	**Mean (SD)**
**Physical Activity Environment**		
**School level measure (possible range)**		
Quality of school physical activity facilities (0-3)	2.6 (0.5)	2.5 (0.4)
Suitability of school grounds for physical activity (3-9)	8.3 (1.5)	8.0 (1.4)
Extra-curricular opportunities for physical activity (0-25)	11.0 (2.2)	12.5 (3.7)
Weekly hours of Physical Education (0+)	2.0 (0.0)	2.2 (.4)
Area around school suitable for physical activity (3-15)	11.9 (2.2)	12.8 (1.2)
School attitude towards physical activity (5-25)	18 (6.0)	19.3 (6.3)
**Recruitment rates**		
Number of Year 9 students at baseline (N)	1648	1759
Recruited at baseline N (%)	1369 (83.1)	1469 (83.5)
**Students from each family affluence group by school-level SEP**	**N (%)**	**N (%)**

For peer review only

Low individual-SEP	266 (19.4)	132 (9.0)
Middle individual-SEP	598 (43.7)	608 (41.4)
High individual-SEP	505 (36.9)	729 (49.6)

Note: Higher scores= more favourable facilities, opportunities, environment or attitude

SEP=Socioeconomic position, SD=Standard deviation

Stage 2: Intervention uptake

Table 2 provides a breakdown recruitment by school-level SEP, suggesting that a lower proportion of students from low-SEP were recruited into the GoActive trial, particularly in high-SEP schools.

Table 3 presents the uptake of the GoActive intervention by individual-level SEP using website engagement. The results show that significantly fewer students of low-SEP than middle- and high-SEP accessed the GoActive intervention website. There was no difference in engagement found for those who did access the website.

Table 3. Website access and engagement of students in the intervention group

	**Low-SEP N=235**	**Middle-SEP N=670**	**High-SEP N=606**	**X^2**	**d.f**	**P-value (adjusted for ties)**
**Accessed the website N (%)**	94 (40.0)	304 (45.4)	315 (52.0)	16.52	2	0.00
**Mean website points (SD)**	49.8 (123.1)	53.2 (85.1)	55.0(87.8)	0.53	2	0.77
**Mean website visits (SD)**	14.2 (28.1)	15.5 (21.0)	16.0 (22.8)	0.74	2	0.69

SEP=Socioeconomic position, SD=Standard deviation

Stage 3: Intervention effect

Table 4 shows the moderating effect of SEP on the effectiveness of the GoActive intervention on average daily minutes of MVPA. The results of the interaction analysis suggest at the post-intervention measurement the intervention effect in students of middle/high-SEP was 4.56

For peer review only

For peer review only - http://bmjopen.bmj.com/site/about/guidelines.xhtml

(95%CI: -9.56 to 0.41) minutes/day less MVPA than students of low-SEP. However, subgroup analyses did not show statistically significant effects in either group.

Table 4. Intervention effect on daily accelerometer assessed MVPA at 14-16 weeks and 10-months post intervention and on anthropometric measures at 10-months post intervention

	**B**	**95% CI**	**P-value**	**Model N**
**14-16 weeks post intervention**				
**MVPA**				
Interaction term				
Intervention*SEP	-4.56	-9.56 to 0.41	0.069	1878
Stratified analysis				
Low-SEP	3.13	-1.27 to 7.54	0.150	241
Middle/High-SEP	-1.49	-6.54 to 3.57	0.540	1637
**10-months post intervention**				
**MVPA**				
Interaction term				
Intervention*SEP	-7.53	-12.89 to -2.17	0.009	1785
Stratified analysis				
Low-SEP	4.90	0.09 to 9.70	0.046	203
Middle/High-SEP	-2.76	-6.78 to 1.26	0.164	1582
**BMI z-score**				
Interaction effect				
Intervention*SEP	0.12	-0.02 to 0.26	0.096	2070
Stratified analysis				
Low-SEP	-0.10	-0.19 to 0.0	0.055	247
Middle/High-SEP	0.03	-0.05 to 0.12	0.413	1823
**Body fat (%)**				
Interaction term				
Intervention*SEP	1.09	-0.63 to 2.81	0.198	1873
Stratified analysis				
Low-SEP	-0.69	-3.17 to 1.78	0.560	216
Middle/High-SEP	0.41	-0.75 to 1.57	0.464	1657
**Waist circumference (cm)**				
Interaction term				
Intervention*SEP	0.73	-0.68 to 2.15	0.287	2089
Stratified analysis				
Low-SEP	-0.71	-1.64 to 1.30	0.808	249
Middle/High-SEP	0.56	-0.17 to 1.30	0.124	1840

Note: All models adjusted for school-level SEP, county and school clustering; MVPA models also adjusted for baseline MVPA

For peer review only

SEP=Socioeconomic position, MVPA= Moderate-to-vigorous physical activity, BMI=Body mass index

Stage 4: Long-term compliance

At 10-months post intervention, the difference in intervention effect increased to -7.53 (95%CI: -12.89 to -2.17) minutes/day MVPA in favour of participants of low-SEP (Table 4). Subsequent stratified analyses showed a positive intervention effect in participants of a low-SEP (4.90; 0.09 to 9.70) but not those of middle/high.

Stage 5: Evaluation participation

Figure 2a shows that questionnaire compliance decreased throughout the intervention across all socioeconomic groups. The figure also shows an association between individual-level SEP and questionnaire compliance (lower compliance among students of low-SEP). Differences in compliance increased with time from T2 to T3 to T4 (**T2:** X^2 = 23.45, p = 0.00; **T3:** X^2 = 15.25, p = 0.00; **T4:** X^2 = 43.88, p = 0.00). Figure 2b shows this trend was also observed for accelerometer compliance (**BL:** X^2 = 8.90, p = 0.02; **T3:** X^2 = 8.12, p = 0.02; **T4:** X^2 = 33.65, p = 0.00).

Stage 6: Health outcomes

Table 4 shows an indication (p=0.09) of a more favourable intervention effect on the BMI z-score in participants of low-SEP, however the interaction term was not statistically significant. No interaction effects were observed for waist circumference or body fat. Subsequent stratified analyses suggest a favourable intervention effect on BMI z-score among adolescents of low-SEP when compared to the control condition (Low SEP: -0.10; 95% CI:-0.19,0.00), but not for those of middle-high-SEP (middle/high: 0.03; 95% CI:-0.05,0.12).

For peer review only - http://bmjopen.bmj.com/site/about/guidelines.xhtml

See Supplementary Table 1 for mean physical activity and anthropometric outcomes by socioeconomic position and randomisation group at each measurement point.

Discussion

Taking a case-study approach we investigated if and how socioeconomic inequities arose during the intervention and evaluation process of a school-based physical activity intervention called GoActive. In doing so, we present a novel approach to analysing young people's physical activity interventions from an equality lens. The findings described below demonstrate the benefit of taking this approach to intervention evaluation, providing insight beyond the main trial analysis.

We discuss our main findings in relation to three key elements: intervention context and engagement (stages 1 & 2), intervention effectiveness (stages 3, 4 & 6) and intervention recruitment and evaluation (stages 2 & 5).

Intervention context and engagement

Our finding that school-level SEP did not appear to influence the school physical activity environment at baseline, contrasts with previous research highlighting socioeconomic inequities in school physical activity provision and resources.[8,33,34] Whilst it is likely this could be the result of the small sample size of included schools (n=16) and resultant limited power to show significant differences, this could also be due to the UK context of GoActive, where national and local policy, such as the Schools Premises Regulations, impose minimum standards for school sports grounds and facilities.[35,36] In addition to extra funding available for low-SEP schools, such a pupil premium funding[24] which could be spent on the provision of physical activity resources and opportunities.

In relation to engagement, significantly fewer adolescents of low-SEP accessed the GoActive website. Of those who did, a graded effect was observed with adolescents of low-SEP engaging the least. One explanation for this, as highlighted in previous research, is that students living in the context of socioeconomic deprivation interact differently with the

For peer review only

school environment (e.g., the use of equipment, fostering of autonomy, competence and relatedness, update of extracurricular opportunities) potentially impacting their engagement with GoActive.[37,38] Furthermore, review evidence reports that adolescents of low-SEP experience multiple barriers to engaging in physical activity interventions, including digital exclusion.[14]

Intervention effectiveness

Despite apparently lower engagement, our exploratory analyses suggest that participants of a low-SEP responded more favourably to GoActive, with a difference in effect of 7.53 minutes/day at 10-months post-intervention to participants of a middle/high-SEP. It may be that students of a low-SEP had a lower engagement with the website but were more engaged with other elements of the intervention that we have no data on. The observed intervention effect of ~5 minutes of MVPA per day may be important for health,[39] and was the targeted effect in main GoActive trial. A similar pattern of effect was also observed for BMI z-score.[23] Overall, these findings support the potential for school-based interventions to reduce inequities in physical activity and obesity. It is possible more deprived students particularly benefited from the chance to try the variety of new activities offered during the GoActive intervention.[8,25] This is especially promising given the stark socioeconomically patterned inequities in overweight and obesity in the UK and other high-income countries. [8,40]

Our choice to treat adolescents of low-SEP as an independent group was based on recent review evidence that their experiences of physical activity notably differ to those of middle- and high-SEP, highlighting the value of looking at them as a separate group.[8] By doing so, our findings add to the main trial moderation analyses where participants of low-and middle-SEP were grouped, suggesting the intervention effect was primarily experienced among low-SEP adolescents. While the approach initially taken was pre-specified and common among existing literature,[23] mainly due to the small sample size of low-SEP groups, these exploratory analyses suggest that important differences in effect may be overlooked when taking this approach.

Intervention recruitment and evaluation

For peer review only - http://bmjopen.bmj.com/site/about/guidelines.xhtml

Recruitment data showed that 14% of those participating in the GoActive trial were of a low-SEP and the majority of these students attended low-SEP schools. In the East of England, data from the Family Resources Survey (2016-2019) reports 19.5% of young people were living in poverty at the time GoActive was delivered.[41] It is possible this is due to the small sample of 16 schools, that are unlikely to be representative of the county. Furthermore, whilst 'living in poverty' is a different indicator of SEP than family affluence, measures of SEP are shown to be highly correlated.[26,42] It is therefore worth considering, when comparing these percentages, that adolescents of low-SEP might be underrepresented in the overall GoActive sample, aligning with evidence that socioeconomically disadvantaged groups are "hard to reach" and recruit into research.[43] Of those recruited into GoActive, inequities in study evaluation measures were observed. These results are consistent with previously reported socioeconomic patterns in response to survey evaluation measures.[44] [10,45] Higher accelerometer non-response has also been reported among socioeconomically deprived children,[46,47] however, there is a lack of research looking at socioeconomic patterning in accelerometer compliance among adolescent populations.

Based on these findings, it is important to acknowledge that our analyses were conducted using a small subset of students of low-SEP which may result in bias in our conclusions. It is possible that differential engagement and response to evaluation measures resulted in a subset of students of low-SEP who were not reflective of the group more broadly, impacting the generalisability of our results. Furthermore, it is possible this may have impacted the results of our analyses under stages 3 and 4, where those who remained in GoActive are more likely to be those who got most out of it.

Strengths and limitations

Previous research has begun to look at differential effectiveness using the primary outcome of the intervention.[9] To our knowledge, this is the first paper to provide an example of how inequities can be explored throughout the intervention and research process of young people's physical activity interventions. Taking a stage-based approach we highlight differential engagement in specific components of the GoActive intervention, including

For peer review only

accessing the GoActive website and in response to evaluation measures. We further highlight the potential of school-based interventions to reduce inequities in MVPA and obesity. Further strengths include the diversity of data collected during the GoActive trial which allowed us to build a more holistic picture of inequities during the trial and the use of device-measured MVPA, which aligns with public health research recommendations for the objective and comprehensive evaluation of health promotion programmes.[25,48]

Whilst presenting our result as exploratory, rather than confirmatory, we acknowledge the small sample size of the low-SEP group and school-level data raises problems with regards to statistical power.[49] The subjective quantification of school environment features may have given rise to self-report biases.[37] It is possible that teachers' reported acceptability of physical activity resources was relative to school-level deprivation, with teachers at high-SEP schools expecting a higher standard of resources and facilities. It is also suggested that some activity types (e.g. biking, stair walking) and intensities can be misclassified by wrist-worn accelerometers.[50] If these behaviours are also socioeconomically patterned, this may have led to an under or overestimation of the difference in effect between subgroups. It is also possible that differential access to computers outside of school hours may have impacted engagement with the GoActive website.[14] Further limitations include the relative lack of participants of a low-SEP and of non-white ethnicity.[25]

It is acknowledged that this is an exploratory post-hoc study of the main trial data collected for GoActive. It is therefore presented as an example of one approach to exploring intervention generated inequities throughout the intervention and evaluation process. With this in mind, the operationalisation of each stage (Figure 1) and the resultant analyses were based on the best available data from the GoActive trial and not what would ideally be the most appropriate data to address each stage of Love's model. Data were not available to address other relevant questions, such as whether schools has access to facilities specifically needed to run GoActive (rather than general facilities) (to address stage 1), the SEP of schools that agreed to participate vs those who did not (to address Stage 2), the role the intervention development process could have played in the uptake of and engagement with the intervention (to address stage 5) or the cumulative effects of inequities across the stages of Love's model. A further limitation was not being able to use the focus group and interview

For peer review only - http://bmjopen.bmj.com/site/about/guidelines.xhtml

data collected as part of the GoActive process evaluation, as information was not available on the SEP of the participants involved. To properly address each stage of Love's model, the stages need to be considered and embedded in the research design.

Recommendation for future research and practice

As highlighted above, this paper presents a case-study example of how to analyse young people's physical activity interventions with an equity lens. Drawing on the stages developed by Love (2019), a framework for future studies to apply, adapt and develop is provided.[22] Whilst the paper focuses on young people's physical activity interventions, the application of this approach more broadly is encouraged. The data required for such a comprehensive analysis should be considered during the design stage of future interventions and trials. This will help prevent the development and implementation of unequitable interventions, making better use of public resources.[51] The financial and resource requirement for running sufficiently large trials to detect a main intervention effect and differential effects between subgroups are acknowledged.[9] To tackle this, Love et al., (2017) have previously recommended encouraging coordinated efforts towards fewer, high-quality, large trials, adequately powered to address questions of differential effectiveness.[9] This study echoes this statement, continuing to solely add evidence on overall effectiveness will continue to limit the evidence-base and our understanding from progressing.

Mobilising the approach presented in this project for existing intervention strategies will further help develop our understanding of why current interventions appear to be ineffective in tackling physical inactivity during adolescence.[12] In addition to developing our understanding of the most appropriate data to address each stage of the model, going forward it would be useful to apply this approach to a range of trials to provide researchers and public health professionals with further examples of how to assess inequities at each stage, generating ideas within the research community and continuing to develop this approach.

The results of this stage-based analysis show the potential for universal school-based physical activity interventions to positively impact socioeconomically deprived students (who remained participating in the trial), reducing inequities. Importantly this contradicts the

For peer review only - http://bmjopen.bmj.com/site/about/guidelines.xhtml

common assumption that interventions generate or exacerbate inequities.[9] The results also demonstrate how intervention components that require individual agency, such as accessing the GoActive website, can exacerbate inequities.[52,53] This should be considered in the development and implementation of school policy, especially in schools with a high proportion of students of a low-SEP. Due to the exploratory nature of this study, it would be beneficial for future research to further study the potential benefit of school-based physical activity interventions for students of low-SEP. It may be useful to explore the application of easily accessible interventions, such as the Daily Mile, to a secondary school setting.[54]

Recruiting and retaining participants of a low-SEP can be challenging, which means they are often underrepresented in research.[14] To increase the reach of interventions and to be able to conduct statistically powered subgroup analyses, the development of active and targeted recruitment of adolescents living in the context of socioeconomic deprivation is an important area for future research. The lack of representation of low-SEP groups in intervention development is an opportunity for growth within school-based interventions and an important area to be considered in the development and evaluation of interventions. Strategies are also needed to better engage these adolescents in the research process, for example involving them in the design and research process through patient and public involvement (PPI).[55]

Conclusion

This was an exploratory study exploring whether and how socioeconomic inequities might arise throughout a school-based physical activity intervention. We demonstrate how the GoActive trial positively affected the physical activity and BMI of low-SEP students. However, differential engagement in the intervention and response to evaluation measures may have biased these conclusions. The continued development and evaluation of school-based interventions from an equity lens is essential as we move out of the Covid-19 pandemic, where disparities in school-based physical activity were exacerbated.[56]

For peer review only - http://bmjopen.bmj.com/site/about/guidelines.xhtml

*** ***

Contributors

OA conceived the study in collaboration with EvS and HF. KC led the GoActive trial and provide feedback on this secondary analysis of the GoActive data, as did PW.

Competing interests

None.

Funding

Olivia Alliott is funded by the National Institute for Health and Care Research (NIHR) School for Public Health Research (SPHR), Grant Reference Number PD-SPH-2015. Hannah Fairbrother is funded through the NIHR School for Public Health Children, young people & families programme. The work of Esther van Sluijs is supported by the Medical Research Council (grant number MC_UU_00006/5). The GoActive trial was funded by the National Institute for Health Research (NIHR) Public Health Research Programme (https://www.nihr.ac.uk/explore-nihr/funding-programmes/public-healthresearch.htm; award number: 13/90/18) and undertaken under the auspices of the Centre for Diet and Activity Research (CEDAR), a UKCRC Public Health Research Centre of Excellence. The GoActive trial was also supported by NIHR Biomedical Research Centre Cambridge: Nutrition, Diet, and Lifestyle Research Theme (Grant IS-BRC-1215-20014) to KC, EvS. The funders had no role in the GoActive study design, data collection and analysis, decision to publish, or preparation of the manuscript. The views expressed in this article are those of the author(s) and the funders had no role in the design of the study, data analysis, data interpretation or in writing the manuscript.

Data availability statement

The authors do not have the authority to share the data that support the findings of this study and the data are not openly available because of ethical and legal considerations. Non-identifiable data can be made available to bona-fide researchers on submission of a reasonable request to datasharing@mrc-epid.cam.ac.uk. The principles and processes for

For peer review only - http://bmjopen.bmj.com/site/about/guidelines.xhtml

accessing and sharing data are outlined in the [MRC Epidemiology Unit Data Access & Data Sharing Policy].

Ethical approval statement

Ethical approval for the GoActive trial was obtained from University of Cambridge Psychology Research Ethics Committee (PRE.2015.126). No additional approval was sought for this study as it was a secondary data analysis.

References

1. Pratt M, Varela AR, Salvo D, Kohl III HW, Ding D. Attacking the pandemic of physical inactivity: what is holding us back? Vol 54: BMJ Publishing Group Ltd and British Association of Sport and Exercise Medicine; 2020:760-762.
2. Blair SN. Physical inactivity: the biggest public health problem of the 21st century. *British journal of sports medicine.* 2009;43(1):1-2.
3. Corder K, Winpenny E, Love R, Brown HE, White M, Van Sluijs E. Change in physical activity from adolescence to early adulthood: a systematic review and meta-analysis of longitudinal cohort studies. *British journal of sports medicine.* 2019;53(8):496-503.
4. Koivusilta L, Rimpelä A, Rimpelä M. Health related lifestyle in adolescence predicts adult educational level: a longitudinal study from Finland. *Journal of Epidemiology & Community Health.* 1998;52(12):794-801.
5. Koivusilta LK, West P, Vesa Markus Antero S, Nummi T, Rimpelä AH. From childhood socio-economic position to adult educational level–do health behaviours in adolescence matter? A longitudinal study. *BMC public health.* 2013;13(1):1-9.
6. Guthold R, Stevens GA, Riley LM, Bull FC. Global trends in insufficient physical activity among adolescents: a pooled analysis of 298 population-based surveys with 1.6 million participants. *The Lancet Child & adolescent health.* 2020;4(1):23-35.
7. Vander Ploeg KA, Maximova K, McGavock J, Davis W, Veugelers P. Do school-based physical activity interventions increase or reduce inequalities in health? *Soc Sci Med.* 2014;112:80-87.
8. Alliott O, Ryan M, Fairbrother H, Van Sluijs E. Do adolescents' experiences of the barriers to and facilitators of physical activity differ by socioeconomic position? A systematic review of qualitative evidence. *Obes Rev.* 2021.
9. Love RE, Adams J, van Sluijs EMF. Equity effects of children's physical activity interventions: a systematic scoping review. *The international journal of behavioral nutrition and physical activity.* 2017;14(1):134.
10. White M, Adams J, Heywood P. How and why do interventions that increase health overall widen inequalities within populations? *Health, inequality and society*. Bristol: Policy Press 2009.
11. Rose G. Sick individuals and sick populations. 1985. *International journal of epidemiology.* 1985;14(1):32-38.

For peer review only

12. Neil-Sztramko SE, Caldwell H, Dobbins M. School-based physical activity programs for promoting physical activity and fitness in children and adolescents aged 6 to 18. *Cochrane Database of Systematic Reviews.* 2021(9).
13. Venkatraman T, Honeyford K, Costelloe CE, et al. Sociodemographic profiles, educational attainment and physical activity associated with The Daily Mile™ registration in primary schools in England: a national cross-sectional linkage study. *Journal of epidemiology and community health.* 2021;75(2):137-144.
14. Craike M, Wiesner G, Hilland TA, Bengoechea EG. Interventions to improve physical activity among socioeconomically disadvantaged groups: an umbrella review. *Int J Behav Nutr Phys Act.* 2018;15(1):1-11.
15. Frohlich KL, Potvin L. Transcending the known in public health practice: the inequality paradox: the population approach and vulnerable populations. *American journal of public health.* 2008;98(2):216-221.
16. Fernandes M, Sturm R. Facility provision in elementary schools: correlates with physical education, recess, and obesity. *Preventive medicine.* 2010;50:S30-S35.
17. Sallis JF, Conway TL, Prochaska JJ, McKenzie TL, Marshall SJ, Brown M. The association of school environments with youth physical activity. *American journal of public health.* 2001;91(4):618.
18. Beauchamp A, Backholer K, Magliano D, Peeters A. The effect of obesity prevention interventions according to socioeconomic position: a systematic review. *Obes Rev.* 2014;15(7):541-554.
19. Rush E, Reed P, McLennan S, Coppinger T, Simmons D, Graham D. A school-based obesity control programme: Project Energize. Two-year outcomes. *British Journal of Nutrition.* 2012;107(4):581-587.
20. Williams NA, Coday M, Somes G, Tylavsky FA, Richey PA, Hare M. Risk factors for poor attendance in a family-based pediatric obesity intervention program for young children. *Journal of developmental and behavioral pediatrics: JDBP.* 2010;31(9):705.
21. Craig CL, Bauman A, Gauvin L, Robertson J, Murumets K. ParticipACTION: A mass media campaign targeting parents of inactive children; knowledge, saliency, and trialing behaviours. *Int J Behav Nutr Phys Act.* 2009;6(1):1-7.
22. Love R. *Inequalities in Children's Physical Activity and Interventions*: Centre for Diet and Physical Activity Research, MRC Epidemiology Unit, University of Cambridge2019.
23. Brown HE, Whittle F, Jong ST, Croxson C, Sharp SJ, Wilkinson P. A cluster randomised controlled trial to evaluate the effectiveness and cost-effectiveness of the GoActive intervention to increase physical activity among adolescents aged 13–14 years. 2017;7:e014419.
24. Department of Education GU. Guidance Using pupil premium: guidance for school leaders. Published 18 October 2019; Last updated 1 November 2021.
25. Corder KL, Brown HE, Croxson CH, et al. A school-based, peer-led programme to increase physical activity among 13-to 14-year-old adolescents: the GoActive cluster RCT. 2021.
26. Galobardes B, Lynch J, Smith GD. Measuring socioeconomic position in health research. *British medical bulletin.* 2007;81(1):21.
27. Pardo-Crespo MR, Narla NP, Williams AR, et al. Comparison of individual-level versus area-level socioeconomic measures in assessing health outcomes of children in Olmsted County, Minnesota. *Journal of epidemiology and community health.* 2013;67(4):305-310.

For peer review only

For peer review only - http://bmjopen.bmj.com/site/about/guidelines.xhtml

28. Johnson MRD, Bhopal RS, Ingleby JD, Gruer L, Petrova-Benedict RS. A glossary for the first World Congress on Migration, Ethnicity, Race and Health. 2019;172:85-88.
29. Harrison F, van Sluijs EM, Corder K, Jones A. School grounds and physical activity: Associations at secondary schools, and over the transition from primary to secondary schools. *Health & place.* 2016;39:34-42.
30. Rowlands AV, Olds TS, Hillsdon M, et al. Assessing sedentary behavior with the GENEActiv: introducing the sedentary sphere. 2014.
31. Phillips LR, Parfitt G, Rowlands AV. Calibration of the GENEA accelerometer for assessment of physical activity intensity in children. *Journal of science and medicine in sport.* 2013;16(2):124-128.
32. Schaefer CA, Nigg CR, Hill JO, Brink LA, Browning RC. Establishing and evaluating wrist cutpoints for the GENEActiv accelerometer in youth. *Medicine and science in sports and exercise.* 2014;46(4):826.
33. Hollis JL, Williams AJ, Sutherland R, et al. A systematic review and meta-analysis of moderate-to-vigorous physical activity levels in elementary school physical education lessons. *Preventive medicine.* 2016;86:34-54.
34. Carlson JA, Mignano AM, Norman GJ, et al. Socioeconomic disparities in elementary school practices and children's physical activity during school. *Am J Health Promot.* 2014;28(3_suppl):S47-S53.
35. GOV.UK. The School Premises (England) Regulations 2012. 2012; https://www.legislation.gov.uk/uksi/2012/1943/contents/made. Accessed 30th November 2021.
36. Department of Education GU. Area guidelines for mainstream schools. 2014; https://assets.publishing.service.gov.uk/government/uploads/system/uploads/attachment_data/file/905692/BB103_Area_Guidelines_for_Mainstream_Schools.pdf. Accessed 30 November 2021.
37. Foubister C, Van Sluijs EM, Vignoles A, et al. The school policy, social, and physical environment and change in adolescent physical activity: An exploratory analysis using the LASSO. *PloS one.* 2021;16(4):e0249328.
38. Moore GF, Littlecott HJ, Evans R, Murphy S, Hewitt G, Fletcher A. School composition, school culture and socioeconomic inequalities in young people's health: Multi-level analysis of the Health Behaviour in School-aged Children (HBSC) survey in Wales. *British Educational Research Journal.* 2017;43(2):310-329.
39. Ekelund U, Luan J, Sherar LB, Esliger DW, Griew P, Cooper A. Moderate to vigorous physical activity and sedentary time and cardiometabolic risk factors in children and adolescents. *Jama.* 2012;307(7):704-712.
40. Mayor S. Socioeconomic disadvantage is linked to obesity across generations, UK study finds. British Medical Journal Publishing Group; 2017.
41. Agrawal S, Phillips D. Catching up or falling behind? Geographical inequalities in the UK and how they have changed in recent years. *The Institute for Fiscal Studies.* 2020:p2.
42. Galobardes B, Shaw M, Lawlor DA, Lynch JW, Davey Smith G. Indicators of socioeconomic position (part 1). *Journal of epidemiology and community health.* 2006;60(1):7-12.
43. Bonevski B, Randell M, Paul C, et al. Reaching the hard-to-reach: a systematic review of strategies for improving health and medical research with socially disadvantaged groups. *BMC medical research methodology.* 2014;14(1):1-29.

For peer review only

For peer review only - http://bmjopen.bmj.com/site/about/guidelines.xhtml

44. Turrell G, Patterson C, Oldenburg B, Gould T, Roy M-A. The socio-economic patterning of survey participation and non-response error in a multilevel study of food purchasing behaviour: area-and individual-level characteristics. *Public Health Nutr.* 2003;6(2):181-189.
45. Ligthart KA, Buitendijk L, Koes BW, van Middelkoop M. The association between ethnicity, socioeconomic status and compliance to pediatric weight-management interventions–a systematic review. *Obes Res Clin Pract.* 2017;11(5):1-51.
46. Rich C, Cortina-Borja M, Dezateux C, et al. Predictors of non-response in a UK- wide cohort study of children's accelerometer-determined physical activity using postal methods. *BMJ open.* 2013;3(3):14.
47. Love R, Adams J. Socioeconomic and ethnic differences in children's vigorous intensity physical activity: a cross-sectional analysis of the UK Millennium Cohort Study. 2019;9(5):e027627.
48. All-Party Commission on Physical A. Tackling Physical Inactivity – A Coordinated Approach. 2014.
49. Haas JP. Sample size and power. *American journal of infection control.* 2012;40(8):766-767.
50. Arvidsson D, Fridolfsson J, Borjesson M. Measurement of physical activity in clinical practice using accelerometers. *Journal of internal medicine.* 2019;286(2):137-153.
51. Wight D, Wimbush E, Jepson R, Doi L. Six steps in quality intervention development (6SQuID). *Journal of epidemiology and community health.* 2016;70(5):520-525.
52. Coggon J, Adams J. 'Let them choose not to eat cake...': Public health ethics, effectiveness and equity in government obesity strategy. *Future Healthcare Journal.* 2021;8(1):49.
53. Adams J, Mytton O, White M, Monsivais P. Why are some population interventions for diet and obesity more equitable and effective than others? The role of individual agency. *PLoS medicine.* 2016;13(4):e1001990.
54. Marchant E, Todd C, Stratton G, Brophy S. The Daily Mile: Whole-school recommendations for implementation and sustainability. A mixed-methods study. *PloS one.* 2020;15(2):28.
55. McDonagh JE, Bateman B. 'Nothing about us without us': considerations for research involving young people. *Archives of Disease in Childhood-Education and Practice.* 2012;97(2):55-60.
56. Ng K, Cooper J, McHale F, Clifford J, Woods C. Barriers and facilitators to changes in adolescent physical activity during COVID-19. *BMJ open sport & exercise medicine.* 2020;6(1):e000919.

FIGURE TITLES

Figure 1. Intervention stages and accompanying research questions explored throughout the study

Based on the model developed by Love (2019).[22]

For peer review only

For peer review only - http://bmjopen.bmj.com/site/about/guidelines.xhtml

874 **Figure 2. Compliance to study evaluation measures throughout the GoActive trial by**
875 **individual level-SEP, indicated by (a) percentage of students proving questionnaire data;**
876 **and (b) percentage of students providing accelerometer data**

For peer review only

For peer review only - http://bmjopen.bmj.com/site/about/guidelines.xhtml

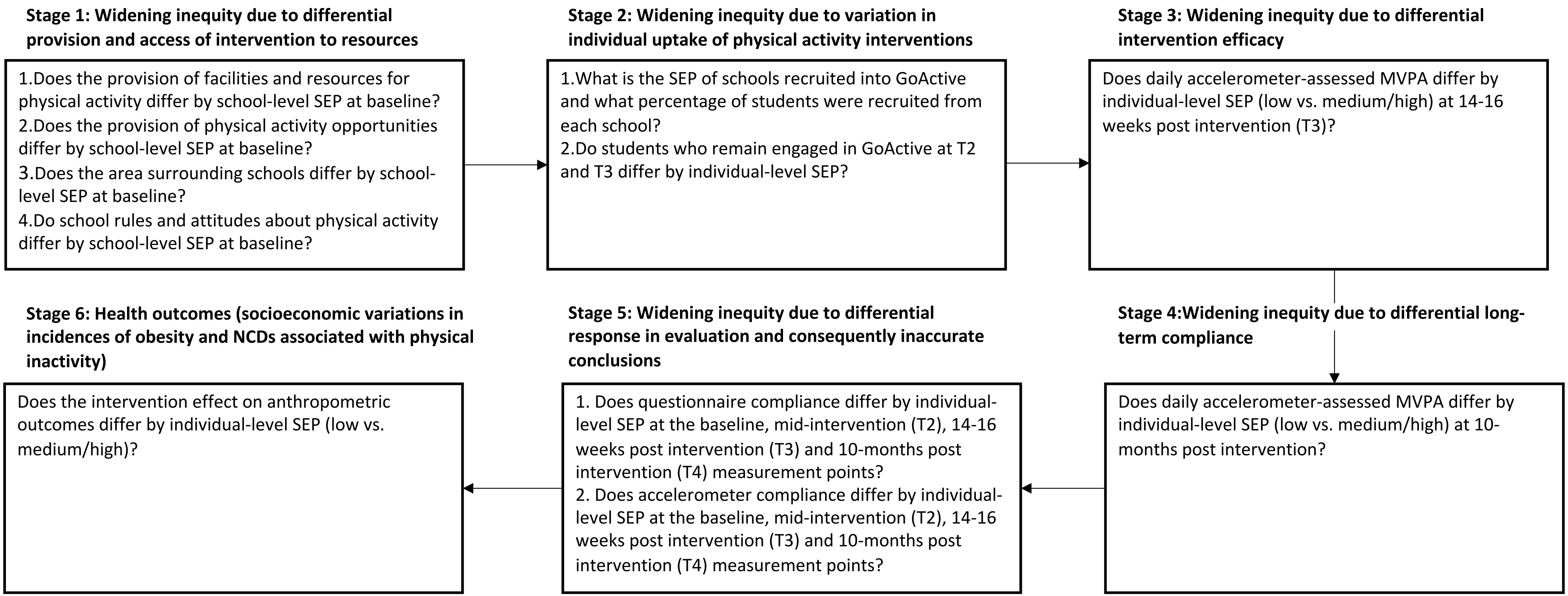
Stage 1: Widening inequity due to differential provision and access of intervention to resources
1.Does the provision of facilities and resources for physical activity differ by school-level SEP at baseline?
2.Does the provision of physical activity opportunities differ by school-level SEP at baseline?
3.Does the area surrounding schools differ by school-level SEP at baseline?
4.Do school rules and attitudes about physical activity differ by school-level SEP at baseline?
Stage 2: Widening inequity due to variation in individual uptake of physical activity interventions
1.What is the SEP of schools recruited into GoActive and what percentage of students were recruited from each school?
2.Do students who remain engaged in GoActive at T2 and T3 differ by individual-level SEP?
Stage 3: Widening inequity due to differential intervention efficacy
Does daily accelerometer-assessed MVPA differ by individual-level SEP (low vs. medium/high) at 14-16 weeks post intervention (T3)?
Stage 4:Widening inequity due to differential long-term compliance
Does daily accelerometer-assessed MVPA differ by individual-level SEP (low vs. medium/high) at 10-months post intervention?
Stage 5: Widening inequity due to differential response in evaluation and consequently inaccurate conclusions
1. Does questionnaire compliance differ by individual-level SEP at the baseline, mid-intervention (T2), 14-16 weeks post intervention (T3) and 10-months post intervention (T4) measurement points?
2. Does accelerometer compliance differ by individual-level SEP at the baseline, mid-intervention (T2), 14-16 weeks post intervention (T3) and 10-months post intervention (T4) measurement points?
Stage 6: Health outcomes (socioeconomic variations in incidences of obesity and NCDs associated with physical inactivity)
Does the intervention effect on anthropometric outcomes differ by individual-level SEP (low vs. medium/high)?

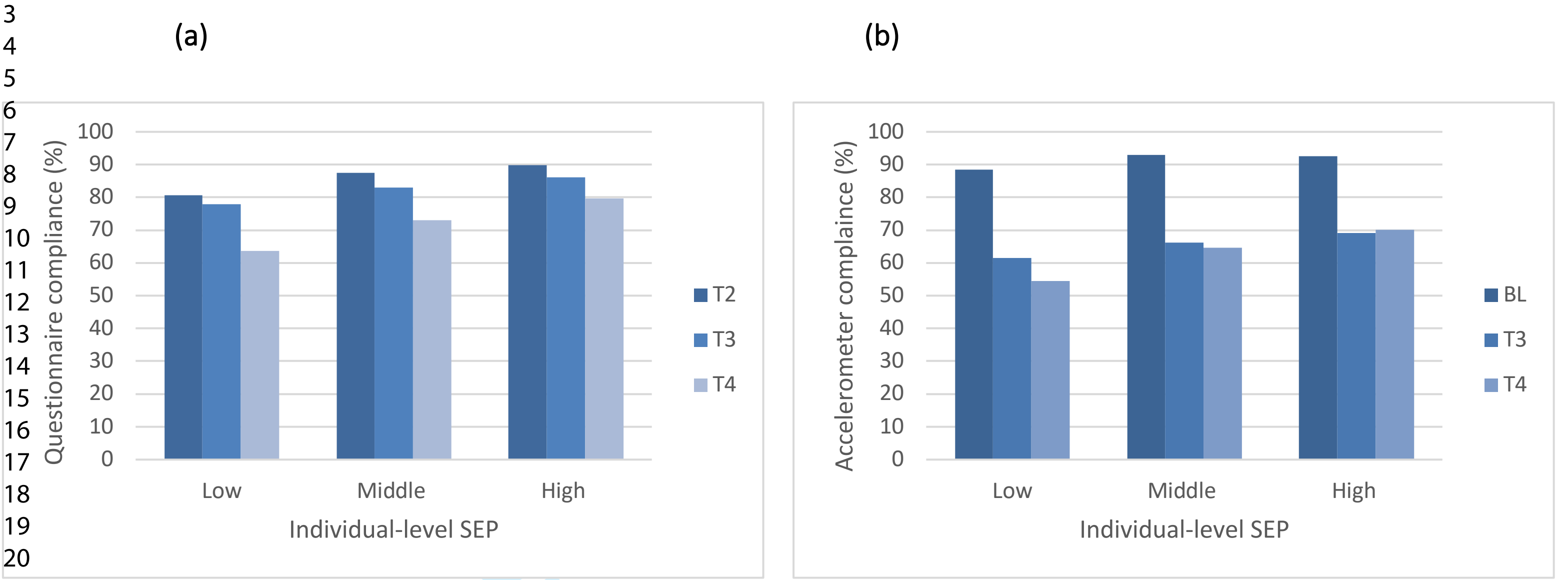

SEP=Socioeconomic position

Supplementary Table 1: Mean physical activity and anthropometric outcomes by socioeconomic position and randomisation group at each measurement point

	Baseline		14-16 weeks post intervention		10-months post intervention	
	Intervention	Control	Intervention	Control	Intervention	Control
**Low-SEP** *Mean (SD)*						
MVPA (minutes)	34.1 (15.4)	36.0 (20.9)	35.6 (21.0)	36.2 (21.7)	29.7 (24.0)	27.4 (17.5)
BMI z-Score	0.26 (2.06)	0.41 (1.71)			0.32 (1.93)	0.64 (1.33)
Body fat (%)	22.0 (9.6)	22.3 (11.1)			18.5 (12.8)	20.6 (12.9)
Waist circumference (cm)	71.2 (10.2)	72.5 (12.5)			72.4 (14.5)	74.8 (12.3)
**Middle/high-SEP** *Mean (SD)*						
MVPA (minutes)	35.9 (18.7)	35.5 (18.7)	33.3 (22.3)	35.4 (21.4)	25.0 (21.0)	27.7 (20.9)
BMI z-Score	0.10 (1.90)	0.17 (1.63)			0.10 (1.88)	0.15 (1.76)
Body fat (%)	20.7 (9.9)	20.5 (9.9)			18.2 (11.7)	18.6 (11.5)
Waist circumference (cm)	70.2 (9.5)	69.7 (9.1)			70.4 (14.3)	70.7 (13.1)

SEP=Socioeconomic position, SD=Standard deviation, MVPA=Moderate-to-vigorous physical activity, BMI=Body mass index

For peer review only
